# SIP1 participates in regulation of flowering time in rice by recruiting OsTrx1 to *Ehd1*

**DOI:** 10.1111/nph.15122

**Published:** 2018-04-03

**Authors:** Pengfei Jiang, Shiliang Wang, Han Zheng, Hao Li, Fei Zhang, Yanhua Su, Zuntao Xu, Haiyan Lin, Qian Qian, Yong Ding

**Affiliations:** ^1^ CAS Center for Excellence in Molecular Plant Sciences School of Life Sciences University of Science & Technology of China Hefei Anhui 230027 China; ^2^ School of Life Sciences Anhui Agricultural University Hefei Anhui 230036 China; ^3^ Key Laboratory of Rice Genetic Breeding of Anhui Province Rice Research Institute Anhui Academy of Agricultural Sciences Hefei 230031 China; ^4^ State Key Laboratory of Rice Biology China National Rice Research Institute Chinese Academy of Agricultural Sciences Hangzhou 310006 China

**Keywords:** C2H2 zinc finger protein SIP1, flowering time, H3K4me3, OsTrx1, rice

## Abstract

- Flowering time (heading date) in rice (*Oryza sativa*) is an important agronomic trait that determines yield. The levels of histone H3 lysine 4 trimethylation (H3K4me3) modulated by TRITHORAX‐like proteins regulate gene transcription, flowering time and environmental stress responses. However, plant TRITHORAX‐like proteins have no known DNA‐binding domain, and therefore the mechanism that gives sequence specificity to these proteins remains unclear.
- Here, we show that the rice TRITHORAX‐like protein OsTrx1 is recruited to its target, *Early heading date 1* (*Ehd1*), by the C2H2 zinc finger protein SDG723/OsTrx1/OsSET33 Interaction Protein 1 (SIP1).
- SIP1 binds to the promoter of *Ehd1* and interacts with OsTrx1. Mutations in *SIP1* led to a late heading date under long‐day and short‐day conditions. Defects in OsTrx1 or SIP1 led to reduced H3K4me3 levels at *Ehd1*, thus reducing *Ehd1* expression.
- Together, our results show that the transcription factor SIP1 interacts with OxTrx1, allowing OsTrx1 to specifically target *Ehd1*, altering H3K4me3 levels, increasing *Ehd1* expression and thereby promoting flowering.

## Introduction

The timing of flowering, the transition from vegetative to reproductive development in plants, is determined by genetic pathways that integrate endogenous and environmental signals (Simpson & Dean, 2002; Izawa, 2007; Lee & An, 2015). Rice (*Oryza sativa*) flowering time (termed heading date) is an important agronomic trait for regional and seasonal adaptation, and heading at the proper time is a critical step for successful grain production (Izawa, 2007; Yeang, 2013; Sun *et al*., 2014). Rice has two homologs of *Arabidopsis thaliana FLOWERING TIME LOCUS T* (*FT*): *Heading date 3a* (*Hd3a*) and *RICE FLOWERING TIME LOCUS T1* (*RFT1*). *Hd3a* and *RFT1* encode proteins that function as florigens under short‐day (SD) and long‐day (LD) conditions, respectively (Yano *et al*., 2001; Kojima *et al*., 2002; Tamaki *et al*., 2007; Komiya *et al*., 2008, 2009). Hd3a and RFT1 are generated in the leaf phloem and transferred to the shoot apical meristem (SAM), where they induce reproductive development (Komiya *et al*., 2008; Hagiwara *et al*., 2009; Lee & An, 2015).

The positive and negative regulators of the florigen genes *Hd3a* and *RFT1* form a network that regulates flowering in rice (Komiya *et al*., 2009). Upstream of *Hd3a* and *RFT1*, rice has a conserved flowering pathway, the *Heading date1* (*Hd1*) pathway, which is controlled by photoperiod (Komiya *et al*., 2008; Takahashi *et al*., 2009). *Hd1*, a homolog of *CONSTANS* (*CO*), promotes flowering under SD by the activation of *Hd3a* and represses flowering under LD by the suppression of *Hd3a* (Yano *et al*., 2000; Ishikawa *et al*., 2005, 2011), respectively.

In addition to the *Hd1* pathway, rice has a unique, independent flowering time pathway mediated by *Early heading date 1* (*Ehd1*). *Ehd1* encodes a B‐type response regulator that promotes flowering by controlling the expression of *FT‐*like genes under SD and LD (Doi *et al*., 2004). *Ehd1*, a central integrator involved in flowering time, is activated by *Ehd2/OsID1* (*OsINDETERMINATE 1*)*/RID1* (*Rice INDETERMINATE 1*) and *Ehd4* under LD and SD conditions (Matsubara *et al*., 2008; Park *et al*., 2008; Gao *et al*., 2013). *OsMADS50* promotes *Ehd1* by suppressing the expression of (*LEAFY COTYLEDON 2 and FUSCA 3‐LIKE 1*) *OsLFL1*, which encodes an inhibitor of *Ehd1* under LD conditions (Lee *et al*., 2004; Ryu *et al*., 2009). OsMADS56 interacts with OsMADS50 *in vitro*; these two MADS‐box proteins form a heterodimer and function antagonistically through the *OsLFL1*–*Ehd1* pathway under LD (Ryu *et al*., 2009). OsMADS51, another MADS‐box gene, promotes *Ehd1* expression under SD (Kim *et al*., 2007). In addition to being activated by MADs‐box proteins, *Ehd1* is suppressed by *Grain Number Plant Height and Heading date7* (*Ghd7*), which encodes a CCT domain protein (Xue *et al*., 2008). *Ghd7* is activated by *EL1* (*Early flowering 1*)/*Hd16*, which encodes a casein kinase I protein (Dai & Xue, 2010), and is suppressed by *Ehd3* (Matsubara *et al*., 2011).

In eukaryotic cells, dynamic changes in chromatin structure mediated by covalent modifications of DNA or histones frequently correlate with changes in transcription. The methylation of histone lysines, including histone H3 lysine 4 (H3K4), H3K9, H3K27, H3K36, H3K79 and H4K20, plays a crucial role in the regulation of key biological processes, such as cell cycle progression, transcription and DNA repair (Zhang & Reinberg, 2001; Liu *et al*., 2010). Except for H3K79 methylation, which is catalyzed by Dot1 family proteins, all other histone lysine methylations are carried out by SET (Su (var), Enhancer of Zeste and Trithorax) domain‐containing enzymes (Takahashi *et al*., 2011). The SET domain family contains yeast Set1 and its orthologs in other species (Avramova, 2009). The TRITHORAX (TRX) family of proteins, including Drosophila Trithorax (Trx), mammalian Mixed lineage leukemia protein 1‐4 (MLL1‐4) and ARABIDOPSIS HOMOLOG of TRITHORAX 1‐2 (ATX1‐2), form a phylogenetic subgroup distinct from yeast Set1. Yeast SET1 is the sole methyltransferase acting on lysine 4 of histone H3, and is responsible for the mono, di‐ and tri‐methylation of H3K4 (Bernstein *et al*., 2002; Santos‐Rosa *et al*., 2002). Arabidopsis ATX1 tri‐methylates H3K4 at specific genes involved in multiple biological processes, such as flowering time regulation, dehydration stress responses and flower organ development (Alvarez‐Venegas *et al*., 2003; Saleh *et al*., 2007; Pien *et al*., 2008; Ding *et al*., 2011a,b, 2012a,b). In addition to development, ATX1 and WDR5a are also associated with RNA polymerase II enrichment, which promotes the transcription initiation at the promoter region and transcription elongation at the gene body (Ding *et al*., 2011b, 2012b). However, ATX2, a homolog of ATX1, has di‐methyltransferase activity (Saleh *et al*., 2008).

The promotion of flowering time also requires the methylation of lysine 4 of H3 and lysine 36 of H3, which is mediated by SET‐domain group (SDG) proteins (Sun *et al*., 2014; Jeong *et al*., 2015; Lee & An, 2015; Shi *et al*., 2015). OsTrx1/SDG723/OsSET33, a rice TRITHORAX‐like protein, interacts with Ehd3 to boost flowering by negatively regulating the expression of *Ghd7* (Choi *et al*., 2014). Flowering promoted by OsTrx1 requires OsWDR5a under LD and SD conditions (Jiang *et al*., 2018). SDG701, related to Arabidopsis SDG2/ATRX3, regulates the tri‐methylation of H3K4 at *Hd3a* and *RFT1* (Liu *et al*., 2017). *SDG724*,* SDG725* and *SDG708* encode proteins belong to SET domain family II; members of this family function in H3K36me2/3 modifications (Sun *et al*., 2012; Sui *et al*., 2013; Choi *et al*., 2014; Liu *et al*., 2016). Loss of function of any of these proteins results in late flowering in both LD and SD conditions. SDG724 is required for the deposition of H3K36me2/3 at more flowering gene loci, such as *Ehd3*,* Ehd2*,* OsMADS50*,* Hd3a* and *RFT1*, whereas SDG725 is only required for H3K36me2/3 deposition at *RFT1* (Sui *et al*., 2013; Sun *et al*., 2014). SDG708 is indispensible for H3K36me2/3 at *Hd3a*,* RFT1* and *Ehd1* (Liu *et al*., 2016).

Histone modifications play critical roles at specific developmental stages, and the functions of H3K4 methylation in developmental regulation, transcription and flowering time in Arabidopsis are well known, but less is known about its functions in the monocot plant rice. In rice, over 50% of genes show H3K4 methylation (Zong *et al*., 2013), suggesting that H3K4 methylation has widespread functions in many biological processes. Although these findings hint at the importance of histone modifying proteins in rice, the fundamental question of how these proteins target specific genes to carry out their functions remains to be understood, particularly in plants. In mammalian cells, MLL1 contains a CXXC motif, which helps it to bind directly to DNA (Ayton *et al*., 2004). By contrast, plant TRITHORAX‐like proteins, including ATX1, ATX2 and OsTrx1/SDG723/OsSET33, lack the CXXC motif. The recruitment of plant TRITHORAX‐like proteins to target genes might depend on their interaction with specific transcription factors; however, the mechanisms that provide specificity to plant TRITHORAX‐like proteins remain to be identified.

OsTrx1/SDG723/OsSET33, encoded by LOC_Os09g04890, interacts with Ehd3 to repress the transcription of *Ghd7* (Choi *et al*., 2014). The PHD motif of OsTrx1 binds the histone H3, allowing OsTrx1 to modify chromatin (Choi *et al*., 2014). However, the molecular mechanism of how OsTrx1 targets the flowering time gene and establishes H3K4me3 remains unclear. Here, we report that OsTrx1 interacts with the transcription factor SIP1, which allows OsTrx1 to bind to *Ehd1*. Defects in OsTrx1 and SIP1 result in late heading under LD and SD conditions and both act in the same flowering pathway in rice.

## Materials and Methods

### Materials

The authorities for all of the species under investigation include: University of Science & Technology of China and Anhui Agricultural University. The plants used in the study are in the *Oryza sativa* ssp. *japonica* cv Nipponbare background. All plants were regenerated from callus as described previously (Xu *et al*., 2015, 2016). T_1_ plants were generated from the seeds of self‐pollinated T_0_ plants. For mutants generated by CRISPR/Cas9, the CRISPR/Cas9 constructs were segregated out in self‐pollinated lines. All rice plants were grown in fields from 20 April to the beginning of October at Hefei (Anhui) in 2016 and 2017, and from 20 November to the beginning of February at Lingshui (Hainan), China in 2016 and 2017. The details of day length for rice growth in Hefei and Lingshui are described in Supporting Information Table S1. All plants were re‐grown in a growth room at 26°C under a restricted LD photoperiod (14 h : 10 h, light : dark cycle) or a restricted SD photoperiod (10 h : 14 h, light : dark cycle). *Arabidopsis thaliana* ecotype WS plants were grown at 22°C under an LD photoperiod (16 h : 8 h, light : dark cycle). Seeds from the *atx1‐1* mutant have been described previously (Alvarez‐Venegas *et al*., 2003; Ding *et al*., 2011a,b).

### Plasmid constructs

The plasmids were constructed using the DNA primers and protocols described in Table S2. All cloned DNA was confirmed by DNA sequencing.

### Yeast one‐hybrid assay

Yeast one‐hybrid assay was performed following the ‘Matchmaker Gold Yeast One‐Hybrid Library Screening System User Manual’ (Clontech, Mountain View, CA, USA). Briefly, the different parts of the *Ehd1* promoter were cloned into the pAbAi vector and SIP1 was fused with pGADT7 (AD‐SIP1). Then, the proEhd1‐pAbAi vectors were transferred into the yeast strain and grown on synthetic defined medium lacking uracil (Ura). The minimal inhibitory concentration of Aureobasidin A (AbA) for the bait strain was tested on medium lacking Ura. Then, AD‐SIP was transferred into bait strains containing different proEhd1‐pAbAi vectors. The protein–DNA interactions were examined on medium lacking leucine (Leu) and Ura with 250 ng ml^−1^ AbA.

### Yeast two‐hybrid assay

The yeast two‐hybrid assay was performed according to the manufacturer's protocol (Clontech, user's manual 630489). Briefly, the *Saccharomyces cerevisiae* strain AH109 was transformed with the bait construct pGBKT7‐OsTrx1, and then transformed with the cDNA library generated from rice leaves by Clontech. pGBKT7‐OsTrx1, pGBKT7‐OsTrx1N or pGBKT7‐OsTrx1C was then co‐transformed with pGADT7‐SIP1. Vectors lacking coding region insertions were used as negative controls. The yeast was scored for protein interaction based on its ability to grow on synthetic defined medium lacking tryptophan (Trp), Leu, histidine (His) and adenine. The primers used to generate the constructs are shown in Table S2.

### Protein pull‐down assays, co‐immunoprecipitation (Co‐IP) and immunoblot assays

For the pull‐down assays, beads were incubated with 3 μg of fusion protein, washed and incubated with 3 μg of soluble protein overnight at 4°C. Mock controls included extracts prepared from either the His‐Tag or glutathione transferase (GST) vectors. The beads were washed five times with a solution containing 20 mM Tris (pH 7.4), 150 mM NaCl and 0.05% Tween 20, separated on a sodium dodecylsulfate‐polyacrylamide gel electrophoresis (SDS‐PAGE) gel and analyzed by immunoblotting using an anti‐GST antibody (GenScript, Nanjing, China; A00866‐100, lot: 13D000626) or an anti‐His antibody (Abmart, Shanghai, China; M30111M, lot: 273884).

For Co‐IP, SIP1 was fused with FLAG tag and OsTrx1 was fused with HA tag, and cloned into the pUC19 vector. Co‐IP was performed as described previously (Lu *et al*., 2017). Briefly, 1 × 10^6^ rice protoplasts were lysed with PEN‐140 buffer (140 mM NaCl, 2.7 mM KCl, 25 mM Na_2_HPO_4_, 1.5 mM KH_2_PO_4_, 0.01 mM EDTA and 0.05% CA‐630). The supernatant was pre‐cleared with Protein G and precipitated with anti‐HA (Sigma‐Aldrich; H9658, lot: 095M4778V) antibodies. The protein complexes were isolated by binding to Protein G beads, followed by five washes with PEN‐400 buffer (400 mM NaCl, 2.7 mM KCl, 25 mM Na_2_HPO_4_, 1.5 mM KH_2_PO_4_, 0.01 mM EDTA and 0.05% CA‐630). The samples were analyzed by immunoblotting using anti‐FLAG (Sigma‐Aldrich; H6908, lot: SLBQ7119V) and anti‐HA antibodies.

### Complementation of the *ostrx‐2* and *atx1* mutants

For *ostrx1‐2* complementation, the *Pro*_*OsTrx1*_ *:FLAG‐OsTrx1* construct was generated by fusing the full‐length cDNA of *OsTrx1* to *FLAG*, and then inserting the fusion into a pCambia1300 vector harboring the 3100‐bp *OsTrx1* promoter. The construct was introduced into the *Agrobacterium tumefaciens* strain *EHA105* and plants were regenerated from callus as described previously (Xu *et al*., 2015).

For *atx1* complementation, the *Pro*_*35S*_ *:RFP‐OsTrx1* construct was generated by fusing the full‐length cDNA of *OsTrx1* to *RFP*, and then inserting the fusion into a pCambia1301 vector. The construct was introduced into the *Agrobacterium tumefaciens* strain *EHA105* and transformed the *atx1* mutant.

### Generation of *ostrx1*,* edh1* and *sip1* mutants using CRISPR/Cas9

The CRISPR/Cas9 mutagenesis was performed as described previously (Xu *et al*., 2015, 2016). Briefly, the oligonucleotides used for *OsTrx1*,* Ehd1* and *SIP1* mutagenesis were designed with the help of CRISPR‐PLANT tools (Yan *et al*., 2015). The oligonucleotides were inserted into the CRISPR/Cas9 vector pHUN4c12 with *Bsa*I. The binary constructs were then introduced into the *Agrobacterium tumefaciens* strain *EHA105* and plants were regenerated from callus. The mutants were further confirmed by sequencing. The oligonucleotides are listed in Table S2.

### Electrophoretic mobility shift assay

The electrophoretic mobility shift assay (EMSA) was performed as described previously (Su *et al*., 2017). Briefly, 1–2 μg of purified protein in binding buffer (10 mM Tris‐HCl, pH 7.5, 100 μM KCl, 1 mM EDTA, 100 μg ml^−1^ bovine serum albumin (BSA), 100 μM ZnCl_2_, 6% glycerol, 1 mM dithiothreitol (DTT)) was mixed with 4 pmol γ‐^32^P ATP‐labeled probe with or without various dosages of unlabeled probe at 4°C for 1 h. After separation in a 4.5% native non‐denaturing acrylamide gel, the gel was exposed to X‐ray film overnight. The sequence of the probe is shown in Table S2.

### Transient expression in rice protoplasts and bimolecular fluorescence complementation

For bimolecular fluorescence complementation (BiFC), *SIP1* and *OsTrx1* were cloned into pUC‐SPYCE (amino acids 156–239) or pUC‐SPYNE (amino acids 1–155). Rice protoplast isolation and transformation were performed as described previously (Zhang *et al*., 2011). Briefly, 7–10‐d‐old rice seedlings were cultured at 26°C on half‐strength Murashige and Skoog (MS) medium under a 12 h : 12 h, light : dark cycle. Stem and sheath tissues were cut into 0.5‐mm strips and immediately transferred into 0.6 M mannitol. After enzymatic digestion, W5 solution (154 mM NaCl, 125 mM CaCl_2_, 5 mM KCl and 2 mM MES at pH 5.7) was added to the sample, followed by resuspension in MMG solution (0.4 M mannitol, 15 mM MgCl_2_ and 4 mM MES, pH 5.7). The protoplasts were co‐transformed with the corresponding constructs and examined under a confocal laser scanning microscope (Zeiss, LSM710) or immunoprecipitated with specific antibodies.

### Promoter β‐glucuronidase (GUS) assay

The vectors containing *GUS* driven by different promoters were transformed into rice protoplasts with or without SIP1. After incubation at 25°C for 12 h, the protoplasts were lysed with lysis buffer (25 mM Tris‐HCl, pH 7.8, 1 mM DTT, 10% glycerol and 1% Triton X‐100). After centrifugation, 100 μl of supernatant was mixed with 900 μl of fluorescent β‐galactosidase (MUG) substrate mix (10 mM Tris‐HCl, pH 8.0, containing 1 mM MUG and 2 mM MgCl_2_). After incubation at 37°C for 30 min, the reaction was stopped with 40 mM Na_2_CO_3_. GUS activity was measured using a fluorometer (HITACHI, U‐281; Thermo Fisher, Fluoroskan Ascent FL, Waltham, MA, USA) with 365 nm excitation wavelength and 456 nm emission wavelength. The luciferase (LUC) activity was measured using the GloMax 96 Luminometer system (Promega, E6501) with LUC mix (Promega, E1980).

### Antibody generation

To generate Anti‐OsTrx1, the N‐terminus of OsTrx1 (amino acids 1–300) was expressed and purified. The antibody was generated by injection of a rat and conducted by Abmart.

To generate anti‐SIP1, the N‐terminus of SIP1 (amino acids 1–100) was expressed and purified. The antibody was generated by injection of rabbits and conducted by GenScript.

### Reverse transcription and qPCR (quantitative real‐time PCR)

Total RNA was isolated from the leaves of 80‐d‐old plants under LD conditions or from those of 45‐d‐old plants under SD conditions, and reverse transcribed with oligo(dT) primers (Promega); the amounts of individual gene transcripts were measured with gene‐specific primers. RT‐PCR analysis was performed with a CFX real‐time PCR instrument (Bio‐Rad) and SYBR Green mixture (Roche). The relative expression of the genes was quantified with the 2^−ΔΔCT^ calculation, using *UBIQUITIN* as the reference housekeeping gene for the expression analyses. The enrichment of DNA at specific genes was quantified with the 2^−ΔΔCT^ calculation, using *UBIQUITIN* as the reference housekeeping gene for chromatin immunoprecipitation (ChIP) assays (see Table S2).

### ChIP assay

ChIP assay was performed as described previously (Lu *et al*., 2017; Su *et al*., 2017). Briefly, 3 g of 80‐d‐old plants was ground and fixed with 1% formaldehyde for 10 min and quenched in 0.125 M glycine. The grounds were extracted in buffer I (0.4 M sucrose, 10 mM Tris (pH 8.0), 5 mM β‐mercaptoethanol, 0.1 mM phenylmethylsulfonylfluoride (PMSF) and protease inhibitor cocktail) and filtered through Miracloth. After centrifugation, the pellet was extracted with buffer II (0.25 M sucrose, 10 mM Tris (pH 8.0), 10 mM MgCl_2_, 1% Triton X‐100, 5 mM β‐mercaptoethanol, 0.1 mM PMSF and protease inhibitor cocktail) and then with buffer III (1.7 M sucrose, 10 mM Tris (pH 8.0), 10 mM MgCl_2_, 1% Triton X‐100, 5 mM β‐mercaptoethanol, 0.1 mM PMSF and protease inhibitor cocktail). The nuclei were then lysed in lysis buffer (50 mM Tris, pH 8.0, 10 mM EDTA, 1% SDS, 5 mM β‐mercaptoethanol, 0.1 mM PMSF and protease inhibitor cocktail) and the extract was sonicated to fragment the DNA to a size range of 300–500 bp. After centrifugation, the supernatant was diluted using dilution buffer (1.1% Triton X‐100, 1.2 mM EDTA, 16.7 mM Tris (pH 8.0), 167 mM NaCl, 0.1 mM PMSF and protease inhibitor cocktail) and pre‐cleared with protein A or protein G magnetic beads. The specific antibodies anti‐H3K4me3 (Abcam, Cambridge, UK; ab8580, lot: GR273043‐6) and anti‐FLAG (Sigma‐Aldrich; F3165, lot: SLBQ7119V), or control IgG serum, were added to the pre‐cleared supernatants for an overnight incubation at 4°C. The antibody–protein complexes were isolated by binding to protein A or protein G beads. The washed beads were heated at 65°C for 8 h with proteinase K to reverse the formaldehyde cross‐linking and digest the proteins. The sample was then extracted with phenol/chloroform and the DNA was precipitated in ethanol and resuspended in water. The purified DNA was analyzed by RT‐PCR with the gene‐specific primers shown in Table S2.

### Accession numbers

Sequence data from this article can be found in the GenBank/EMBL data libraries under the following accession numbers: *OsTrx1/SDG723* (Os09g0134500) and *SIP1* (NC_029264).

## Results

### *OsTrx1* rescues the developmental defects caused by the *atx1* mutation

As rice OsTrx1 is closely related to Arabidopsis ATX1, with 56% sequence identity and 70% similarity, we investigated whether OsTrx1 and ATX1 have similar functions. We generated a vector harboring a full‐length cDNA from *OsTrx1* fused with *RFP* driven by the *Cauliflower mosaic virus* (CaMV) *35S* promoter (*Pro*_*35S*_ *:RFP‐OsTrx1*), which we transformed into the Arabidopsis *atx1‐1* mutant. The early flowering and dwarf phenotypes caused by the *atx1* mutation were rescued by *OsTrx1* (Fig. S1), indicating that OsTrx1 might function as an H3K4 methyltransferase during rice development.

### *OsTrx1* is involved in the *Ehd1–RFT1* pathway

To investigate the roles of OsTrx1 in the flowering time network, we used CRISPR/Cas9 genome editing to generate new alleles, which we termed *ostrx1‐2* and *ostrx1‐3*. In these alleles, an adenine and thymine were inserted in exon 1 and exon 3, respectively (Fig. S2a,b), resulting in premature stop codons (Fig. S2b). As described in Choi *et al*. (2014), the *ostrx1* mutants displayed a strong late‐flowering phenotype in Hefei (LDs). The late‐flowering phenotype existed in Lingshui (SD) and restricted SD conditions (Fig. S2c,d), suggesting that *OsTrx1* might be involved in a photoperiod‐independent pathway.

To determine the effect of these mutations, we first examined the transcript levels of the florigen genes *RFT1* and *Hd3a*. We isolated total RNA from leaves of 80‐d‐old plants and subjected it to quantitative RT‐PCR analysis. *RFT1* and *Hd3a* transcript levels exhibited a strong diurnal rhythm in wild‐type plants, whereas they were markedly reduced in *ostrx1‐2* and *ostrx1‐3* plants throughout the day (Fig. S2e).

*RFT1* and *Hd3a* are regulated by *Ehd1* and *Hd1* (Yano *et al*., 2000; Doi *et al*., 2004; Ishikawa *et al*., 2005, 2011; Komiya *et al*., 2008, 2009), respectively. We therefore measured the mRNA levels of *Ehd1* and *Hd1*, finding that the expression of *Ehd1*, but not *Hd1*, was abolished in *ostrx1* plants. *Ghd7*, which acts as a negative regulator of *Ehd1*, was highly induced in *ostrx1* plants. These results were consistent with the results of a previous study (Choi *et al*., 2014).

Next, we examined the expression of *Ehd2*,* Ehd3*,* Ehd4* and *EL1*, finding that these genes were not affected in the *ostrx1* mutants compared with the wild‐type. The SD flowering activator *OsMADS51*, but not *OsMADS50* or *OsMADS56*, was downregulated in *ostrx1* mutants (Fig. S2e). We then examined the transcripts of *Ehd1*,* RFT1*,* Hd3a*,* Hd1* and *Ghd7* in SDs, and found that *Ehd1*,* RFT1* and *Ehd3a*, but not *Hd1* or *Ghd7*, were downregulated in *ostrx1* mutants (Fig. S2f). These results suggest that *OsTrx1* might be involved in regulating the heading date in rice through the *Ehd1*–*RFT1* pathway under LD conditions.

### *OsTrx1* is required for H3K4me3 deposition at *Ehd1*

**Figure 1:**
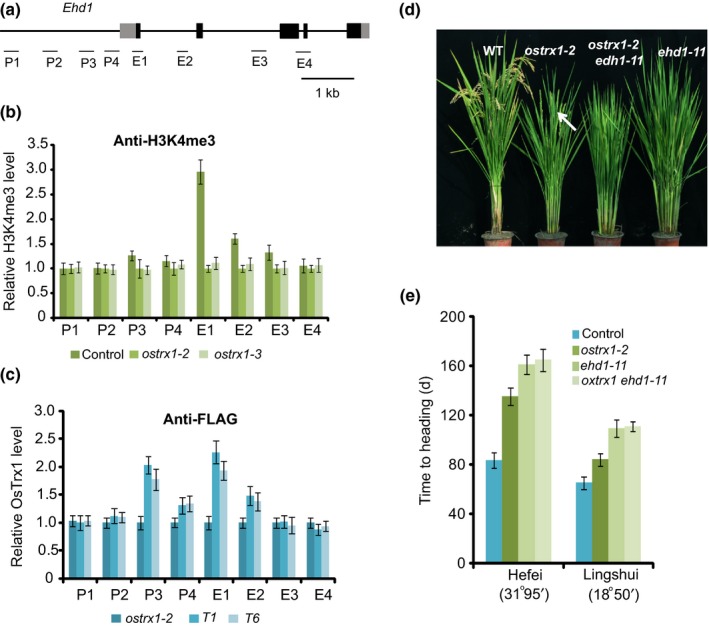
OsTrx1 is required for H3K4me3 at *Ehd1*. (a) Gene structure of *Ehd1*. Exons are indicated by boxes and introns are indicated by lines. The locations of the gene regions analyzed by chromatin immunoprecipitation (ChIP)‐PCR are shown below the diagram. P1–P4 indicate regions in the promoter and E1–E4 indicate regions in the coding region. (b) Relative H3K4me3 levels at different regions of the genes in wild‐type (WT) and *ostrx1* plants. Experiments were repeated at least three times, and the data from the representative experiments shown are presented as means ± SE,* n *=* *3 replicates. (c) Relative OsTrx1 levels in complemented *Pro*_*OsTrx1*_ *:FLAG‐OsTrx1 ostrx1‐2* and *ostrx1‐2* plants. *T1* and *T6* are indicated in Supporting Information Fig. S3(e). Experiments were repeated at least three times, and the data from the representative experiment shown are presented as means ± SE,* n *=* *3 replicates. (d) Representative image of 130‐d‐old WT, *ostrx1‐2*,* ehd1‐11* and *ostrx1 ehd1‐11* plants grown under LD (Hefei). Heading is indicated by an arrow. (e) Days to heading in WT, *ostrx1‐2*,* ehd1‐11* and *ostrx1‐2 ehd1‐11* under long‐day (LD) (Hefei) and short‐day (SD) (Lingshui). Values shown are mean ± standard deviation of heading days; 20 plants were scored per line.

As OsTrx1 rescued the phenotype caused by the *atx1* mutation, we investigated whether the reduction in transcript levels of the genes described above was caused by attenuated H3K4me3 in the *ostrx1* mutants. We investigated the distribution of H3K4me3 by ChIP, followed by quantitative PCR measurement of DNA enrichment. Indeed, H3K4me3 levels were reduced at *Ehd1*,* RFT1* and *Hd3a* in *ostrx1* plants, indicating that OsTrx1 is required for H3K4me3 at these loci (Figs 1a,b, S3a,b). In contrast with *Ehd1*,* RFT1* and *Hd3a*, the transcript levels of *Ghd7* were induced in *ostrx1* mutants (Choi *et al*., 2014) and it was found that the H3K4me3 level of *Ghd7* was upregulated in *ostrx1* plants (Fig. S3c,d).

To examine whether OsTrx1 binds to these loci, we conducted ChIP with OsTrx1. To this end, we generated a construct harboring the full‐length *OsTrx1* sequence fused to a FLAG tag driven by the native *OsTrx1* promoter (*Pro*_*OsTrx1*_ *:FLAG‐OsTrx1*). Transformation with this construct rescued the *ostrx1* late‐flowering phenotype, indicating that FLAG‐OsTrx1 retains OsTrx1 function (Fig. S3e,f). We measured the occupancy of OsTrx1 via ChIP analysis with a specific anti‐FLAG antibody, followed by quantitative PCR. OsTrx1 was enriched along the promoter and coding region of *Ehd1*, but not *RFT1*,* Hd3a or Ghd7* (Figs 1c, S3g,h). These results indicate that OsTrx1 directly targets *Ehd1* and increases the H3K4me3 levels of the *Ehd1* locus.

To elucidate the relationship between *OsTrx1* and *Ehd1*, we generated an *ehd1* mutant using CRISPR/Cas9. In the *ehd1‐11* allele, an adenine was deleted in exon 3 of *Ehd1*, leading to an early stop codon in this mutant (Fig. S4a,b). The *ehd1‐11* plants exhibited a severe late‐heading phenotype (Fig. 1d,e). We then examined the genetic relationship between *OsTrx1* and *Ehd1* by generating the *ostrx1‐2 ehd1‐11* double mutant. The *ostrx1‐2 ehd1‐11* mutant displayed a late‐heading phenotype when grown in Hefei (LD) and Lingshui (SD), similar to the *ehd1‐11* single mutant (Fig. 1d,e), indicating that OsTrx1 is involved in an *Ehd1*‐dependent pathway.

### OsTrx1 interacts with SIP1 *in vitro* and *in vivo*

Given that OsTrx1 has no known DNA‐binding domain, we therefore asked how OsTrx1 could target *Ehd1*. We investigated whether any transcription factors physically interact with OsTrx1 to allow it to target *Ehd1*. We fused OsTrx1 with a binding domain (BD) and screened its potential interactors using a cDNA library with the yeast two‐hybrid system. Sequencing 10 positive colonies from screening revealed that two insertions were in the correct frame and able to encode proteins. One encoded a WD40‐domain protein, and another encoded a protein containing a C2H2‐type zinc finger domain (Fig. S5a), which we named SIP1 (SDG723/OsTrx1 Interaction Protein 1). SIP1 is encoded by LOC_Os09g38790. Phylogenetic analysis revealed that SIP1 is conserved in multiple plant species, including rice, *Setaria italica*,* Brachypodium distachyon*,* Sorghum bicolor*, maize (*Zea mays*) and *A. thaliana*, but not in mammalian cells (Fig. S5b).

**Figure 2:**
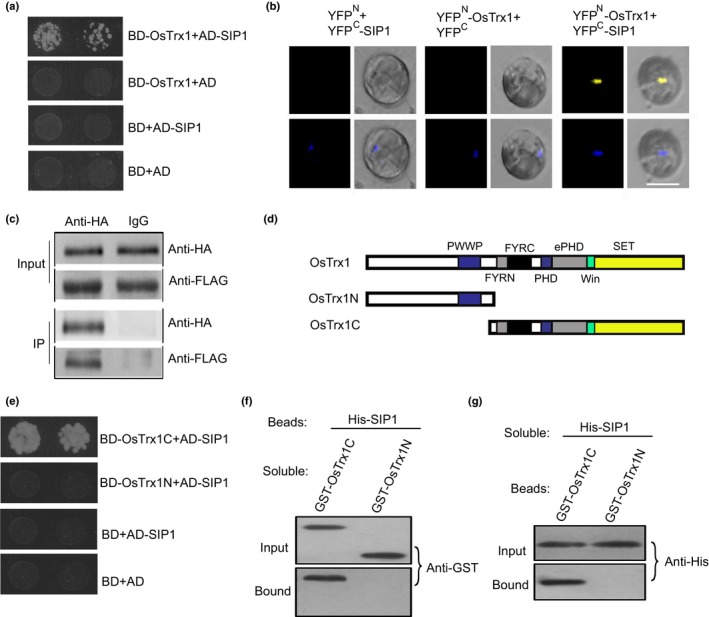
OsTrx1 interacts with SIP1. (a) Yeast two‐hybrid assay revealing an interaction between SIP1 and OsTrx1. The growth of two dilutions (2 × 10^−2^ and 2 × 10^−3^) of yeast culture on synthetic defined (SD) medium lacking Trp, Leu, His and adenine is shown. (b) OsTrx1 fused to the N‐terminus of yellow fluorescent protein (YFP) or the N‐terminus of YFP alone was tested for its ability to bind to the C‐terminus of YFP alone or the C‐terminus of YFP fused to SIP1. Yellow fluorescence and bright field images were recorded and the resulting images were merged. The nucleus was stained by 4′,6‐diamidino‐2‐phenylindole , and appears as a blue dot. Bar, 10 μm. (c) Co‐immunoprecipitation of SIP1 and OsTrx1. *FLAG‐OsTrx1* and *HA‐SIP1* were co‐transformed into rice protoplasts, and the expressed proteins were immunoprecipitated using an anti‐HA antibody and detected with anti‐HA and anti‐FLAG antibodies. IgG was used as a negative control. (d) Diagram of OsTrx1 showing its different domains. (e) The N‐terminus of OsTrx1 and the C‐terminus of OsTrx1 fused to binding domain (BD) were tested for their ability to bind to AD‐SIP1. The growth of two dilutions (2 × 10^−2^ and 2 × 10^−3^) of yeast culture on synthetic defined medium lacking Trp, Leu, His and adenine is shown. (f) Beads containing His‐fused SIP1 were assayed for their ability to bind soluble protein composed of glutathione transferase (GST) fused to the N‐terminus or C‐terminus of OsTrx1. The input and bound proteins were detected with an antibody to GST (anti‐GST). (g) Beads containing the GST‐fused N‐terminus of OsTrx1 or C‐terminus of OsTrx1 were assessed for their ability to bind soluble His‐fused SIP1 and detected with antibody to His (anti‐His).

We further confirmed the OxTrx1–SIP1 interaction by reciprocal yeast two‐hybrid tests, BiFC and Co‐IP experiments. First, we generated a vector harboring full‐length SIP1 fused with AD, and found that OsTrx1 directly bound to SIP1 in yeast (Fig. 2a). For BiFC, functional YFP was observed in the nucleus in cells co‐transformed with OsTrx1‐YFP^N^ (fused with the N‐terminal half of yellow fluorescent protein) and full‐length SIP1‐YFP^C^ (fused with the C‐terminal half of YFP) (Fig. 2b). Immunoprecipitation using HA antibody revealed that OsTrx1 bound to SIP1 in rice protoplasts co‐transformed with *HA‐OsTrx1* and *FLAG‐SIP1* (Fig. 2c).

We then investigated which domain of OsTrx1 is essential for the OxTrx1–SIP1 interaction. Yeast two‐hybrid tests of different fragments of OsTrx1 showed that the C‐terminus of OsTrx1, but not its N‐terminus, bound to SIP1 (Fig. 2d,e). This yeast two‐hybrid interaction was confirmed by a pull‐down assay. Beads attached to SIP1 fused with His successfully bound to the C‐terminus of OsTrx1 fused with GST, but not to the N‐terminus of OsTrx1 fused with GST (Fig. 2f). In the complementary pull‐down assay, the C‐terminus of OsTrx1 fused with GST bound to His‐fused SIP1 (Fig. 2g). These results indicate that OsTrx1 interacts with SIP1 *in vitro* and *in vivo*.

### Loss of *SIP1* function leads to a late heading date

**Figure 3:**
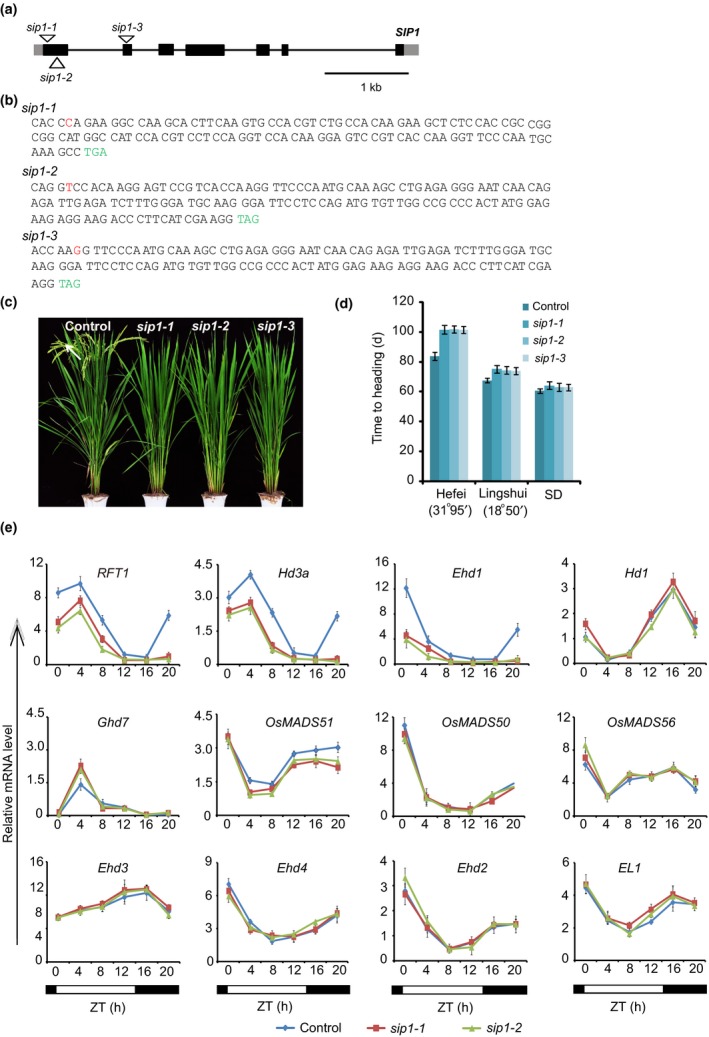
Mutations in *SIP1* result in a late heading date. (a) Gene structure of *SIP1*, indicating exons (boxes), introns (lines) and nucleotide insertions or deletions (triangles). (b) A nucleotide insertion in *sip1‐1* and a nucleotide deletion in *sip1‐2* and *sip1‐3* result in early stop codons in all three mutant alleles. The inserted and deleted nucleotides are marked in red, and stop codons caused by a shift in the open reading frame (ORF) are indicated in green. (c) Representative image of 103‐d‐old wild‐type and *sip1* mutants in long‐day (LD) (Hefei). (d) Days to heading in wild‐type and *sip1* plants under LD (Hefei), short‐day (SD) (Linshui) and restricted SD. Values shown are the mean ± standard deviation of heading days; 20 plants were scored per line. (e) Transcript levels of flowering network genes in *sip1* mutants. RNA isolated from leaves of 80‐d‐old plants under restricted long‐day conditions was used for RT‐PCR. Closed bars, dark period; open bars, light period. ZT, Zeitgeber time. The *y*‐axis shows the transcript level relative to rice *Ubiquitin*. Experiments were repeated at least three times, and the data from the representative experiment shown are presented as means ± SE,* n *=* *3 replicates.

To investigate the genetic function of *SIP1* in regulating the heading date, we generated *sip1* mutants using CRISPR/Cas9. Sequencing revealed that the *sip1‐1*,* sip1‐2* and *sip1‐3* alleles have a cytosine insertion in exon 1, a thymine deletion in exon 1 and a guanine deletion in exon 2, respectively, which result in early stop codons (Fig. 3a,b). All three *sip1* mutants showed a late‐flowering phenotype when grown in Hefei (LD), Lingshui (SD) and restricted SD conditions (Fig. 3c,d), suggesting that *SIP1* might also be involved in a photoperiod‐independent pathway.

We then investigated the expression of genes in the flowering network in the *sip1* mutants. RT‐PCR results revealed that the transcript levels of *Ehd1*,* RFT1*,* Hd3a* and *OsMADS51* were reduced, whereas the transcript level of *Ghd7* was upregulated in the *sip1* mutants compared with the wild‐type. The transcripts of *Hd1*,* Ehd2*,* Ehd3*,* Ehd4*,* OsMADS50*,* OsMADS56* and *EL1* were not affected in the *sip1* mutants (Fig. 3e). These observations are consistent with the late‐flowering phenotype caused by *sip1* mutation and suggest that *SIP1* might be involved in the *Ehd1*–*RFT1* pathway.

**Figure 4:**
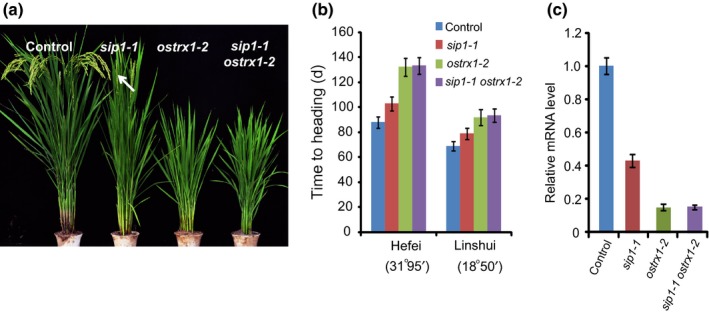
*SIP1* acts in the same flowering time pathway as *OsTrx1*. (a) Representative image of 110‐d‐old wild‐type, *sip1‐1*,* ostrx1‐2* and *sip1‐1 ostrx1‐2* plants in Hefei. Heading is indicated by an arrow. (b) Days to heading in wild‐type, *sip1‐1*,* ostrx1‐2* and *sip1‐1 ostrx1‐2* plants in Hefei and Lingshui. Values shown are the mean ± standard deviation of heading days; 30 plants were scored per line. (c) The transcript level of *Ehd1* in wild‐type, *sip1‐1*,* ostrx1‐2* and *sip1‐1 ostrx1‐2*. The plants were grown under long‐day conditions. RNA was isolated at 20 h after lights‐on Zeitgeber time (ZT20). Experiments were repeated at least three times, and the data from the representative experiment shown are presented as means ± SE,* n *=* *3 replicates.

The possibility of a genetic relationship between *OsTrx1* and *SIP1* was investigated by crossing *sip1‐1* into *ostrx1‐2*. The *sip1‐1 ostrx1‐2* double mutant exhibited a late‐flowering phenotype similar to that of *ostrx1‐2* (Fig. 4a,b). These results suggested that *SIP1* and *OsTrx1* might function together to regulate the same downstream genes. To further confirm these results, we examined the transcripts of *Ehd1* in the *sip1 ostrx1‐2* double mutant. RT‐PCR results revealed that the level of the *Ehd1* transcript in *sip1 ostrx1‐2* was similar to that in *ostrx1‐2* (Fig. 4c). These results indicate that *SIP1* and *OsTrx1* act in the same pathway.

### SIP1 activates *Pro*_*Ehd1*_ *:GUS* activity

**Figure 5:**
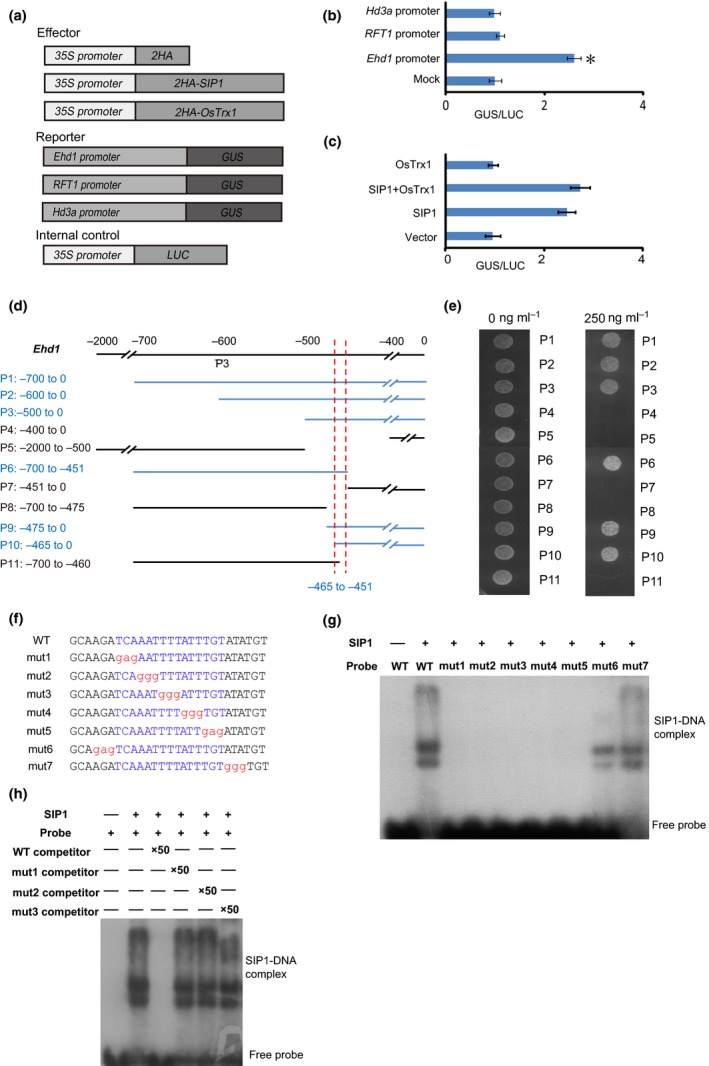
SIP1 binds to the *Ehd1* promoter. (a) Vectors used in the GUS activity assay. (b) β‐Glucuronidase (GUS) activity from the *Pro*_*Ehd1*_ *:GUS*,* Pro* _*RFT*__*1*_ *:GUS* and *Pro*_*Hd3a*_ *:GUS* reporter constructs for cells transformed with *SIP1*. The *x*‐axis shows the relative GUS activity compared with the internal luciferase control (*35S:LUC*). Asterisks indicate *P *<* *0.05 by *t*‐test. (c) GUS activity from the *Pro*_*Ehd1*_ *:GUS* reporter constructs for cells transformed with *SIP1* and *OsTrx1*. The *x*‐axis shows the relative GUS activity compared with the internal luciferase control (*35S:LUC*). Asterisks indicate *P *<* *0.05 by *t*‐test. (d) Diagram of the *Ehd1* promoter showing its different regions. The position of each region is marked on the left. (e) Yeast one‐hybrid assay revealing that 15 base pairs in the *Ehd1* promoter are essential for SIP1 binding. Yeast grown on 0 μM Aureobasidin A (AbA) was used as a negative control. (f) The sequences of probes used for electrophoretic mobility shift assay (EMSA). The conserved motif TCAAATTTTATTTGT is shown in blue, and the mutated nucleotides are shown in red. The motif is located in P3 of Fig. 2(a). (g, h) Gel shift assay using SIP1 and various probes. The ability of SIP1 to bind to different mutated probes labeled with ^32^P was assessed (g), and this binding specificity was tested by adding unlabeled wild‐type competitor probe or mutated probes (h).

We next investigated whether SIP1 directly activates the expression of *Ehd1*,* RFT1* and *Hd3a* by assessing the activation of these genes by SIP1 using reporter constructs harboring *Pro*_*Ehd1*_ *:GUS*,* Pro*_*RFT1*_ *:GUS* and *Pro*_*Hd3a*_ *:GUS*. The vectors containing GUS driven by different promoters and *35S:LUC* were co‐transformed into rice protoplasts with or without SIP. *Pro*_*Ehd1*_ *:GUS* activity, but not *Pro*_*RFT1*_ *:GUS* or *Pro*_*Hd3a*_ *:GUS*, was highly induced in the presence of SIP1 (Fig. 5a,b). OsTrx1 failed to activate *Pro*_*Ehd1*_ *:GUS* activity, but OsTrx1 did not reverse the SIP1‐induced activation (Fig. 5a,c). These observations suggest that SIP1 might activate *Ehd1* via its promoter.

### SIP1 binds to the *Ehd1* promoter

We therefore investigated whether SIP1 directly binds to the *Ehd1* promoter. We fused SIP1 with an AD and used a yeast one‐hybrid system to test whether it interacted with fragments of the *Ehd1* promoter. SIP1 directly binds to the *Ehd1* promoter region from –465 to –451, suggesting that this region is essential for SIP1 binding (Figs 5d,e, S6).

We then investigated the binding specificity of SIP1 via an EMSA. A retarded band was observed when SIP1 was present, whereas the mutated probes showed reduced binding (Fig. 5f,g). The shifted band was abolished when we used a specific competitor probe, but not mutated competitor probes (Fig. 5h), indicating that SIP1 binds to the promoter of *Ehd1 in vitro*. We investigated whether this motif could be found in the promoters of flowering time genes, but this motif was not present in the promoters of genes tested in *sip1* plants.

### SIP1 is required for OsTrx1 occupancy at *Ehd1*

**Figure 6:**
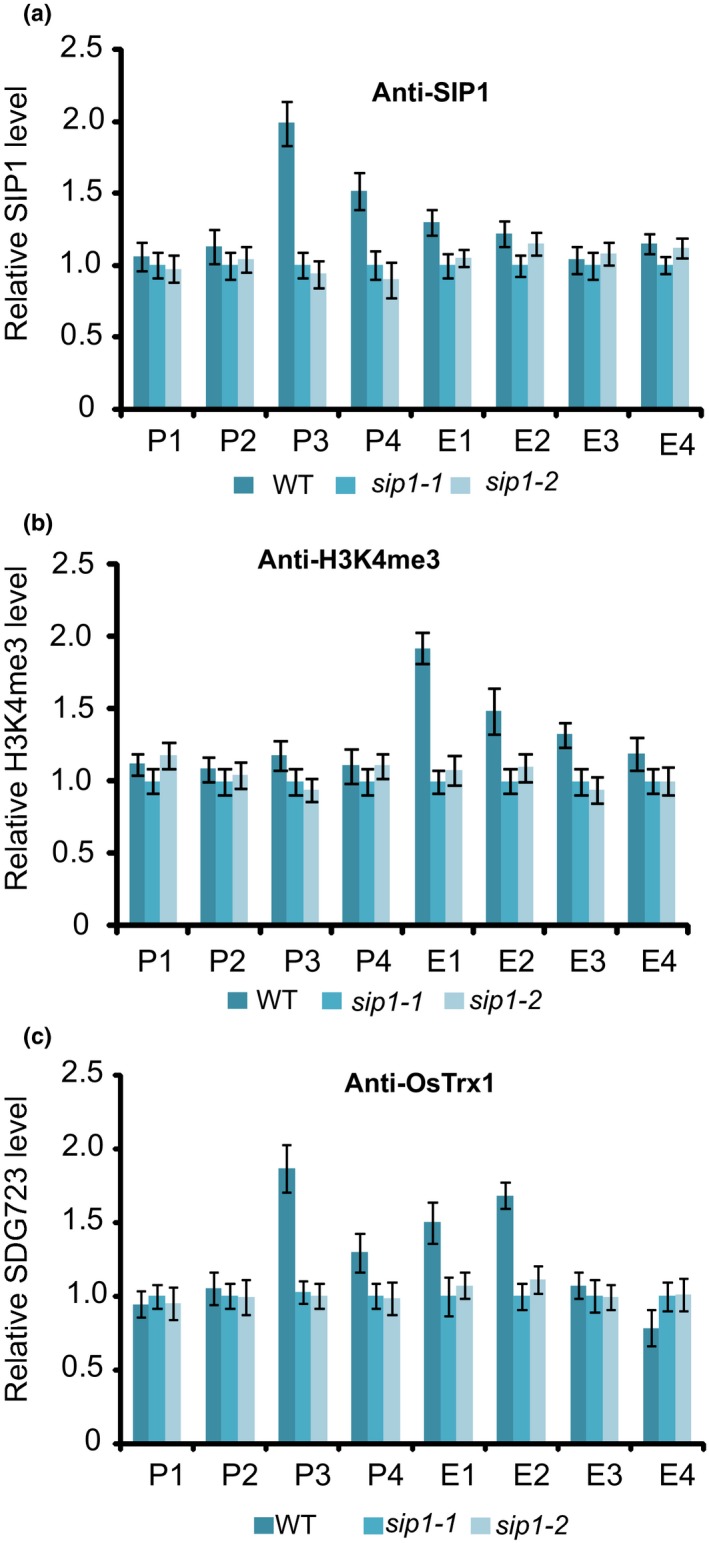
SIP1 is required for OsTrx1 occupancy at *Ehd1*. (a, b) Relative SIP1 and H3K4me3 levels in wild‐type and *sip1* plants. (c) Relative OsTrx1 levels in wild‐type and *sip1* plants. From (a) to (c), experiments were repeated at least three times, and the data from the representative experiments shown are presented as means ± SE,* n *=* *3 replicates. P1–P4 and E1–E4 are indicated in Fig. 1(a).

To investigate whether SIP1 binds to *Ehd1 in vivo*, we generated an antibody specific to SIP1 (Fig. S7a) and assessed the binding of SIP1 to the *Ehd1* promoter using ChIP analysis with the SIP1 antibody, followed by quantitative PCR analysis of DNA enrichment at multiple points along *Ehd1*. SIP1 was highly enriched at the *Ehd1* promoter, but not in *sip1* mutants (Fig. 6a). These results demonstrate that SIP1 can bind to *Ehd1 in vivo*.

As SIP1 binds directly to *Ehd1* and activates its expression, we investigated whether SIP1 is required for OsTrx1 to target *Ehd1*, which, in turn, would affect H3K4me3 levels. We examined H3K4me3 levels by ChIP‐PCR, finding that H3K4me3 of *Ehd1* was reduced in *sip1* (Fig. 6b), suggesting that *SIP1* is required for the maintenance of proper H3K4me3 levels at *Ehd1*.

As OsTrx1 can accomplish H3K4me3 and interacts with SIP1, we then investigated whether SIP1 is required for the binding of OsTrx1 to *Ehd1*. We measured the enrichment of OsTrx1 via ChIP with a specific antibody (Fig. S7b,c), followed by quantitative PCR analysis of DNA enrichment. OsTrx1 enrichment was reduced in the *sip1* mutants compared with the wild‐type, indicating that the targeting of *Ehd1* by OsTrx1 depends on SIP1 (Fig. 6c). We then investigated SIP1 enrichment and found that it was not affected in the *ostrx1* mutant, suggesting that SIP1 binding to *Ehd1* was independent of OsTrx1 (Fig. S7d).

## Discussion

In this study, we have demonstrated that OsTrx1 interacts with the transcription factor SIP1 to modulate flowering, measured as the heading date, in rice. OsTrx1 interacts with SIP1, which allows OsTrx1 to increase H3K4me3 levels at *Ehd1*. Defects in OsTrx1 and SIP1 result in a late heading date under both LD and SD conditions, and these two factors are involved in the *Ehd1–RFT1* pathway. These results were further supported by the finding that defects in SIP1 and OsTrx1 reduce the expression levels and H3K4me3 levels of *Ehd1*. Together, these results provide compelling evidence that SIP1 recruits OsTrx1 to modulate the heading date by regulating the expression level and H3K4me3 level at *Ehd1*.

In mammalian cells, MLL1 directly binds DNA via its CXXC domain. However, no known CXXC domain is present in yeast SET1 and the plant TRITHORAX family proteins ATX1, ATX2 and OsTrx1. ATX1 participates in pre‐initiation complex formation at the promoter and adds the H3K4me3 modification in the gene body (Ding *et al*., 2011b, 2012b; Fromm & Avramova, 2014). ATX1 has multiple domains, including Tudor, DAST, PWWP, ePHD, Win and SET, which have diverse functions. The ePHD domain affects expression from specific ATX1‐dependent genes through its ability to bind the lipid phosphatidyl inositol‐5 phosphate (PtdIns5P), influencing the nuclear cytoplasmic distribution of ATX1 (Alvarez‐Venegas *et al*., 2006; Fromm & Avramova, 2014). In addition, the ePHD domain of ATX1 is associated with lipid binding, which is different from the PHD domain in protein binding (Alvarez‐Venegas *et al*., 2006; Ding *et al*., 2009, 2012c; Fromm & Avramova, 2014). OsTrx1 rescues the developmental defects caused by the *atx1* mutation, suggesting that OsTrx1 and ATX1 have similar functions.

Previous results have suggested that *OsTrx1* promotes flowering via the repression of the expression level of *Ghd7* (Choi *et al*., 2014). The PHD domain of OsTrx1 directly binds to H3 and the C4HC2H motif of OsTrx1 interacts with Ehd3 containing a PHD finger motif. Defects in OsTrx1 might disrupt Ehd3′s ability to repress *Ghd7*, resulting in late flowering (Choi *et al*., 2014). Given that the H3K4me3 level is correlated with transcriptional activation (Ding *et al*., 2011b; Fromm & Avramova, 2014), the induced transcription levels and reduced H3K4me3 levels of *Ghd7* suggest that *Ghd7* might not be the direct target of OsTrx1. A recent study has indicated that OsWDR5a interacts with the Win motif of OsTrx1 and directly binds to histone H3 (Jiang *et al*., 2018). Plants in which *OsWDR5a* expression is decreased by RNA interference produce fewer secondary branches and less grain, and exhibit a delayed heading date under LD and SD conditions, whereas loss of *OsWDR5a* function results in embryo lethality. OsWDR5a binds to *Ehd1* to regulate its H3K4me3 and expression levels (Jiang *et al*., 2018), which is consistent with our current study.

Our study indicates that OsTrx1 interacts with the C2H2 zinc finger protein SIP1. SIP1 binds to the promoter of *Ehd1* and recruits OsTrx1 to accomplish H3K4me3 at *Ehd1*. In Arabidopsis, transcriptional factor VAL1, interacting with LHP1 and binding to *FLOWERING LOCUS C* (*FLC*), results in the mediation of H3K27 trimethylation at *FLC* (Qüesta *et al*., 2016; Yuan *et al*., 2016). The transcription factor SUF4 interacts with MOS1 (MODIFIER OF snc1) to regulate the flowering time (Li *et al*., 2010; Bao *et al*., 2014). Recent results have supported the idea that transcriptional factors are critical for histone phosphorylation to regulate flowering time and hypocotyl elongation in Arabidopsis (Su *et al*., 2017; Zheng & Ding, 2018; Zheng *et al*., 2018).

In *A. thaliana*, a model LD plant, control of floral transition by endogenous (autonomous, gibberellin, circadian clock, age, sugar budget) and environmental (vernalization, ambient temperature, photoperiod) cues is a key factor in adaptation to different regions (Izawa, 2007; Bluemel *et al*., 2015). The floral repressor FLC and the floral promoter CONSTANS (CO) control the florigen gene *FT*; FLC and CO are key transcriptional regulators of the flowering pathways. Mutations in *ATX1* lead to early flowering as a result of reduced transcript levels of *FLC* (Pien *et al*., 2008; Jiang *et al*., 2009).

Rice, a model SD plant, contains two major flowering pathways: a conserved photoperiod pathway and a unique photoperiod‐independent flowering time pathway. *Ehd1* is an evolutionarily unique gene in rice that does not have an ortholog in Arabidopsis. The regulation of *Ehd1* expression also involves a gene network distinct from that in Arabidopsis (Doi *et al*., 2004; Tsuji *et al*., 2011). Ehd1 promotes the expression of *RFT1*, and *Ehd1* transcript levels increase in the presence of *OsGI*,* Ehd2*,* Ehd3* and *Ehd4* under LDs. By contrast, Ehd1 promotes the expression of *Hd3a*, and *Ehd1* transcript levels increase in the presence of *OsGI* under SD conditions (Doi *et al*., 2004; Komiya *et al*., 2009; Tsuji *et al*., 2011). In the current study, OsTrx1 and SIP1 were identified as new factors that positively regulate *Ehd1*, suggesting that the activation of *Ehd1* requires factors involved not only in transcriptional regulation, but also in histone modification. Together, our findings indicate that SIP1 interacts with OsTrx1, which, in turn, regulates the expression of *Ehd1* and heading date in rice.

## Author contributions

Y.D. and P.J. conceived the study and designed the experiments. P.J. performed most of the experiments. S.W. and H.Z. performed EMSA. H.Li performed the rice transformation. F.Z. performed the ChIP‐PCR. Y.S. and Z.X. prepared the materials. H.Lin and Q.Q. performed the bioinformatics analysis. All authors took part in the interpretation of the results and preparation of the manuscript. Y.D. wrote the manuscript. P.J. and S.W. contributed equally to this work.

## Supporting information

Please note: Wiley Blackwell are not responsible for the content or functionality of any Supporting Information supplied by the authors. Any queries (other than missing material) should be directed to the *New Phytologist* Central Office.

**Fig. S1** Complementation of *atx1‐1* with *OsTrx1*.
**Fig. S2** Generation of *ostrx1* mutants using CRISPR/Cas9.
**Fig. S3** H3K4me3 profiles at *RFT1*,* Hd3a* and *Ghd7*.
**Fig. S4** Generation of the *ehd1* mutant using CRISPR/Cas9.
**Fig. S5** *SIP1* encodes a C2H2 zinc finger protein.
**Fig. S6** Identification of SIP1 binding sites in the *Ehd1* promoter.
**Fig. S7** The specificity of the antibodies for SIP1 and OsTrx1.
**Table S1** Average daylight in 10‐day intervals in 2017 at Hefei and Lingshui, China
**Table S2** The constructs and primers used in this study
