# Supplementary material for: SIP1 participates in regulation of flowering time in rice by recruiting OsTrx1 to Ehd1
Source: New Phytol. 2018 Apr 3;219(1):422–35. doi: 10.1111/nph.15122 (PMC6001661; doi:10.1111/nph.15122)
Supplement: Supplementary file 1 — Fig. S1 Complementation of atx1‐1 with OsTrx1. Fig. S2 Generation of ostrx1 mutants using CRISPR/Cas9. Fig. S3 H3K4me3 profiles at RFT1, Hd3a and Ghd7. Fig. S4 Generation of the ehd1 mutant using CRISPR/Cas9. Fig. S5 SIP1 encodes a C2H2 zinc finger protein. Fig. S6 Identification of SIP1 binding sites in the Ehd1 promoter. Fig. S7 The specificity of the antibodies for SIP1 and OsTrx1. Table S1 Average daylight in 10‐day intervals in 2017 at Hefei and Lingshui, China Table S2 The constructs and primers used in this study [file NPH-219-422-s001.pdf]

**New Phytologist Supporting Information:** Figs S1–S7 and Tables S1 & S2

**Article title:** SIP1 participates in regulation of flowering time in rice by recruiting OsTrx1 to Ehd1

**Authors:** Pengfei Jiang, Shiliang Wang, Han Zhenga, Hao Lic, Fei Zhanga, Yanhua Sua, Zuntao Xua, Haiyan Lind, Qian Qiand, and Yong Ding

**Article acceptance date:** 20 February 2018

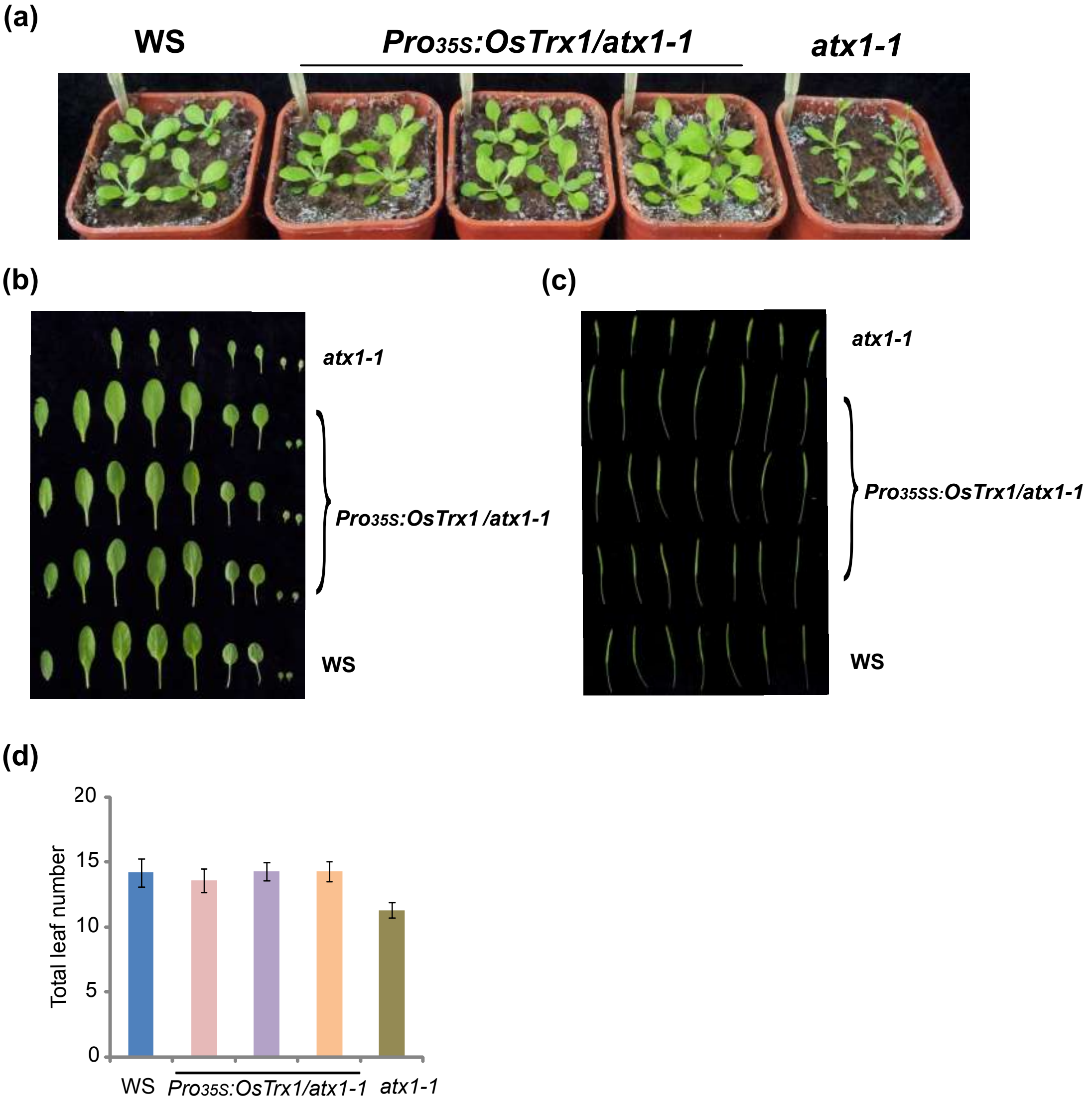


**Fig. S1. Complementation of *atx1-1* with *OsTrx1*.**

The early flowering of *atx1-1* was complemented in lines harboring *OsTrx1* (a); the phenotypes of leaves and siliques of wild-type, and complemented plants are shown in (b) and (c); the total leave number of wild-type, complemented plants, and *atx1-1* is shown in (D). Values shown are mean ± standard deviation of heading days; 20 plants were scored per line.

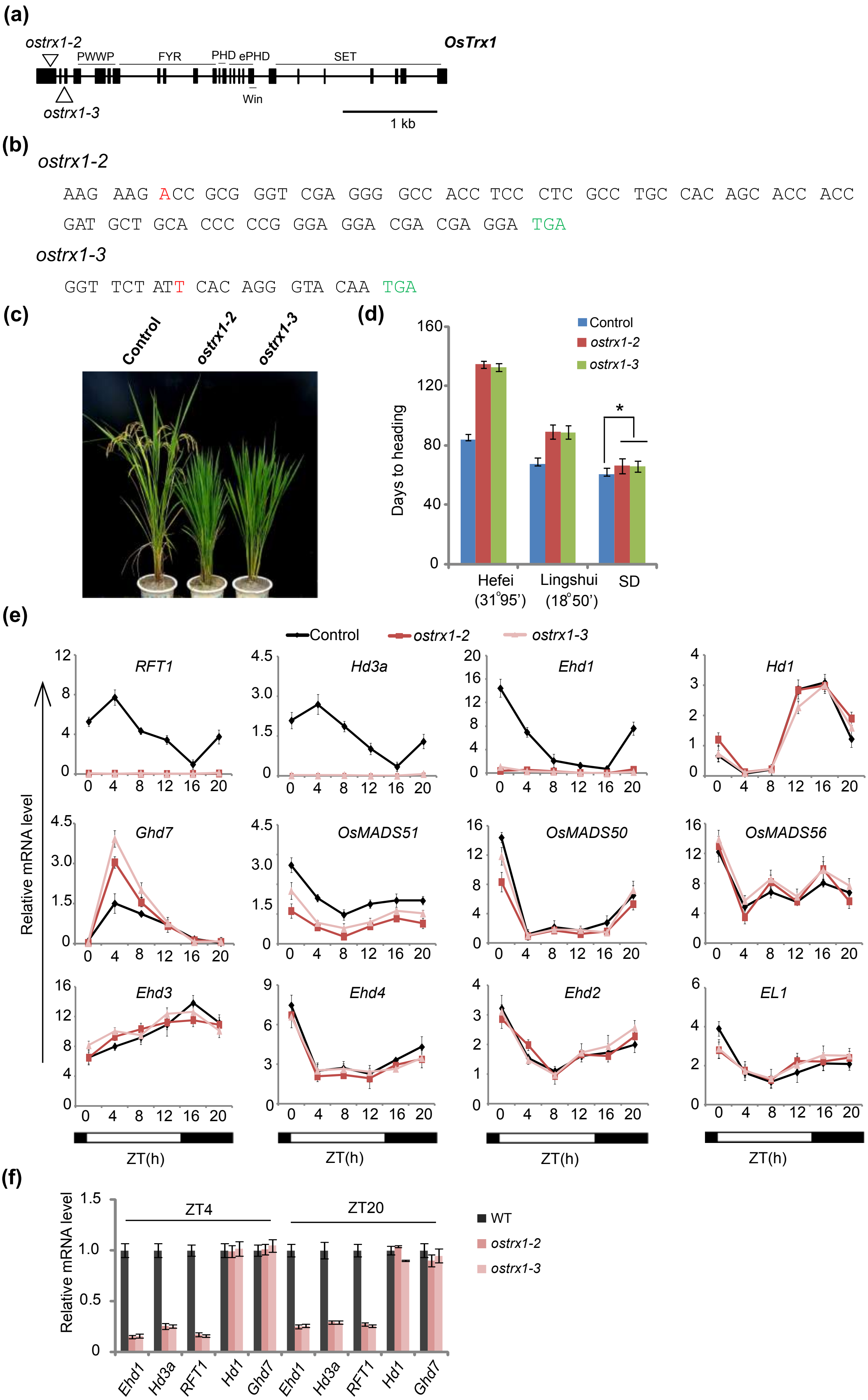
(a)
ostrx1-2
PWWP
FYR
PHD ePHD
SET
OsTrx1
Win
ostrx1-3
1 kb
(b)
ostrx1-2
AAG AAG ACC GCG GGT CGA GGG GCC ACC TCC CTC GCC TGC CAC AGC ACC ACC
GAT GCT GCA CCC CCG GGA GGA CGA CGA GGA TGA
ostrx1-3
GGT TCT ATT CAC AGG GTA CAA TGA
(c)
Control
ostrx1-2
ostrx1-3
(d)
Control
ostrx1-2
ostrx1-3
Days to heading
Hefei (31°95')
Lingshui (18°50')
SD
(e)
Control
ostrx1-2
ostrx1-3
Relative mRNA level
RFT1
Hd3a
Ehd1
Hd1
Ghd7
OsMADS51
OsMADS50
OsMADS56
Ehd3
Ehd4
Ehd2
EL1
ZT(h)
(f)
Relative mRNA level
ZT4
ZT20
WT
ostrx1-2
ostrx1-3
Ehd1
Hd3a
RFT1
Hd1
Ghd7

**Fig. S2. Generation of *ostrx1* mutants using CRISPR/Cas9.**

(a) Gene structure of *Ostrx1* showing exons (boxes), introns (lines), and nucleotide insertions (triangles).

(b) Nucleotide insertions in *ostrx1-2* and *ostrx1-3* result in an early stop codon. The inserted nucleotides are shown in red, and the stop codons caused by a shifted open reading frame (ORF) are indicated in green.

(c) Representative image of 120-day-old wild type and *ostrx1* plants in Hefei (LD).

(d) Days to heading of wild-type and *ostrx1* plants in Hefei, Lingshui, and under restricted short-day conditions. Asterisks indicate $P < 0.05$ by t-test.

(e) Transcript levels of flowering network genes in *ostrx1* mutants under long-day conditions.

RNA isolated from leaves of 80-day-old plants under restricted long-day conditions was used for RT-PCR. The black bars indicate the dark period, and the white bars indicate the light period. ZT, Zeitgeber time. The y-axis shows the transcript level relative to rice *Ubiquitin* expression. The experiments were repeated at least three times, and each experiment included three replicates, $n = 3$ replicates.

(f) Transcript levels of *Ehd1*, *RFT1*, *Hd3a*, *Hd1*, and *Ghd7* under restricted shot-day conditions.

RNA was isolated at 4 hours after lights-on Zeitgeber time (ZT4) and ZT20, respectively. Experiments were repeated at least three times, and the data from the representative experiment shown are presented as means ± SE, $n = 3$ replicates.

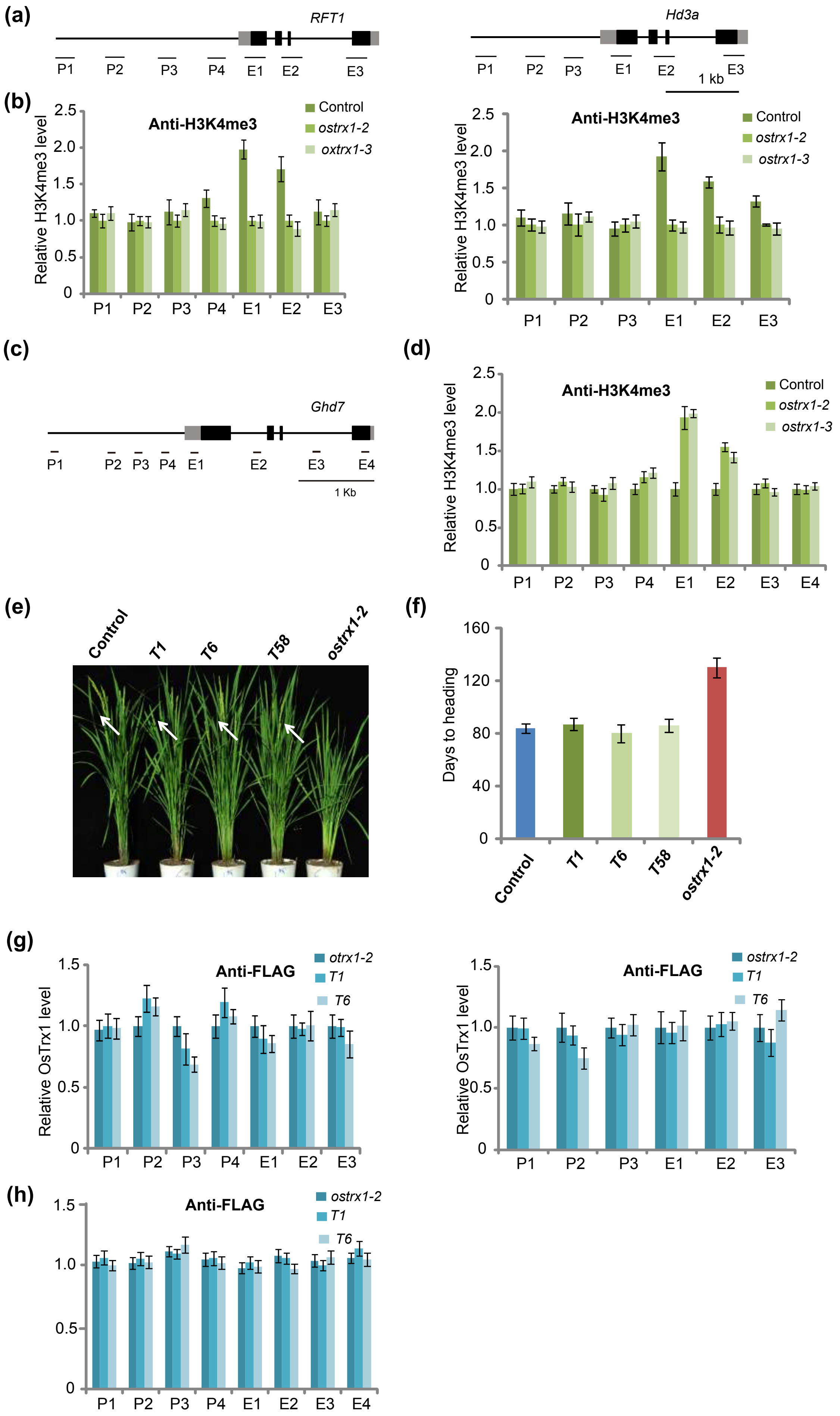

(a)
RFT1
P1 P2 P3 P4 E1 E2 E3
Hd3a
P1 P2 P3 E1 E2 E3
1 kb
(b)
Anti-H3K4me3
Control
ostrx1-2
oxtrx1-3
Relative H3K4me3 level
2.5 2.0 1.5 1.0 0.5 0
P1 P2 P3 P4 E1 E2 E3
Anti-H3K4me3
Control
ostrx1-2
ostrx1-3
Relative H3K4me3 level
2.5 2.0 1.5 1.0 0.5 0
P1 P2 P3 E1 E2 E3
(c)
Ghd7
P1 P2 P3 P4 E1 E2 E3 E4
1 Kb
(d)
Anti-H3K4me3
Control
ostrx1-2
ostrx1-3
Relative H3K4me3 level
2.5 2.0 1.5 1.0 0.5 0
P1 P2 P3 P4 E1 E2 E3 E4
(e)
Control T1 T6 T58 ostrx1-2
(f)
Days to heading
160 120 80 40 0
Control T1 T6 T58 ostrx1-2
(g)
Anti-FLAG
otrx1-2
T1
T6
Relative OsTrx1 level
1.5 1.0 0.5 0
P1 P2 P3 P4 E1 E2 E3
Anti-FLAG
ostrx1-2
T1
T6
Relative OsTrx1 level
1.5 1.0 0.5 0
P1 P2 P3 E1 E2 E3
(h)
Anti-FLAG
ostrx1-2
T1
T6
1.5 1.0 0.5 0
P1 P2 P3 P4 E1 E2 E3 E4

**Fig. S3. H3K4me3 profiles at *RFT1, Hd3*, and *Ghd7.***

(a) Gene structures of *RFT1* and *Hd3a.* Exons are indicated by boxes and introns are indicated by lines. The locations of the gene regions analyzed by ChIP-PCR are presented below the diagrams.

(b) Relative H3K4me3 levels at different regions of *RFT1* (left panel) and *Hd3a* (right panel) in wild-type and *ostrx1* plants. Experiments were repeated at least three times, and the data from the representative experiment shown are presented as means ± SE, *n* = 3 replicates..

(c) Gene structures of *Ghd7.* Exons are indicated by boxes and introns are indicated by lines. The locations of the gene regions analyzed by ChIP-PCR are presented below the diagrams.

(d) Relative H3K4me3 levels at different regions of *Ghd7* in wild-type and *ostrx1* plants. Experiments were repeated at least three times, and the data from the representative experiment shown are presented as means ± SE, *n* = 3 replicates.

(e) Representative image of 90-day-old wild type, three complemented plants harboring $Pro_{OsTrx1}$*: FLAG-OsTrx1* (*T1*, *T6*, and *T58*), and *ostrx1-2* mutants under LD (Hefei) conditions.

(f) Days to heading of wild-type, three complemented plants, and *ostrx1* plants under LD (Hefei) conditions.

(g) Relative OsTrx1 levels at *RFT1* (left panel) and *Hd3a* (right panel) in plants complemented by $Pro_{OsTrx1}$*: FLAG-OsTrx1* and *ostrx1-2*. Experiments were repeated at least three times, and the data from the representative experiment shown are presented as means ± SE, *n* = 3 replicates.

(h) Relative OsTrx1 levels at *Ghd7* in plants complemented by $Pro_{OsTrx1}$*: FLAG-OsTrx1* and *ostrx1-2*. Experiments were repeated at least three times, and the data from the representative experiment shown are presented as means ± SE, *n* = 3 replicates.

(a)

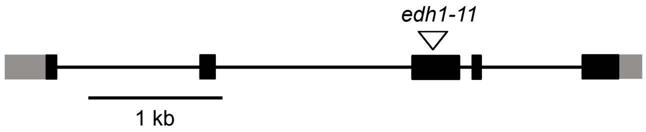


(b)

*ehd1-11*

TTC CTG CTA AAC CTG TGA

**Fig. S4. Generation of the *ehd1* mutant using CRISPR/Cas9.**

(a) Gene structure of *Ehd1*, indicating exons (boxes), introns (lines), and nucleotide insertion (triangles).

(b) A nucleotide insertion in *ehd1-11* results in an early stop codon. The inserted nucleotide is indicated in red, and stop codons caused by the shift of the open reading frame (ORF) is indicated in green.

**(a)**

MGKKKKRVEKVFCYYCDREFDDEKILVQHQKAKHFKCHVCHKKLSTAGGMAIHVLQVH

Zinc Finger motif

KESVTKVPNAKPERESTEIEIFGMQGIPPDVLAAHYGEEEDPSSKVAKVEVPSLRPPVMP
NPAGMVYPPRPAYGVAPPMYNPALNPLMARPPIWPAPPPQPWFTQPVVSVPQMASGL
APQQPLFPIQNMPAPMTSAPANLLQTSFPMAHVGVPSPVTPQVSQPLFPVSTSAGNGA
VSSPYVASVAPGSIPTSSPSVAPAGVGYAATNQGTGGPAAVPPPASNNKAPATQPGANE
VYLVWDDEAMSMEERRLSLPKYQVHDETSQMNSVDAAIDRRISESRLAGRMAL

**(b)**

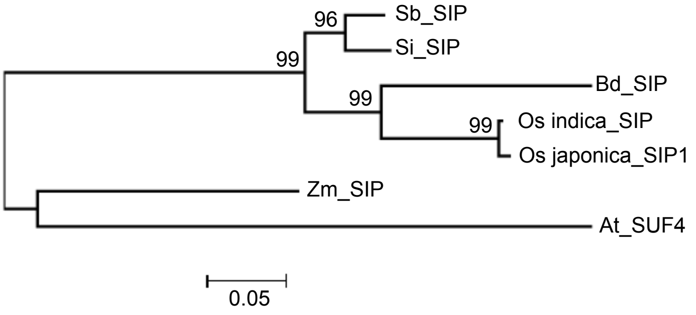


**Fig. S5.** ***SIP1*** **encodes a C2H2 zinc figurer protein.**

(a) The SIP1 protein sequence is shown and the zinc finger motif is underlined.

(b) Phylogenetic analysis of SIP1 from different species. SIP1 sequences from rice, *Setaria italica*, *Brachypodium distachyon*, sorghum (*Sorghum bicolor*), maize (*Zea mays*), and *Arabidopsis thaliana* were aligned with ClustalW. The relationships of the sequences were examined with MEGA5.

**Supplemental Figure 6**

ATTCCTCAATGCATTGCCAGCCAGCCTATCCAGACAAGGCTAGATAATTTGGCTTTTATATCTTCATGTTTAATTT
ATTTTTGGCATTGCGATGGATTTTGATATCTCATTTACCGCAATAGCTTTGAGACATTTTGGCGGTTGGCAAGCTA
TATTTCTTTCAATCTATATATATTGCAAACGTCAATATCGTTGTAATGTGAGAATATAAATAATTGGGAGAAACAT
TAAAGTTGGATAATATAATAATTTATCTAACTGTGTAATTCAATCTGTAAAACAAACTATAATGACTATAAGTTTA
ATTTAATTTCTACATTAACTTAAAAGATAAGCACAACACGCAACATATCACCGTAACACACAAACTGGGTCTTAGC
TATTATTTATCATTAAGATCCTTTTCAAAAAACTGCTACTATAAAAATTCTGAAGATATAATTAAAGTGTGGTTAC
CAAGAACAAGGACACACTAGCATCTCGATCAAACGGCTACTACCTCCGTCCTAAAATGTAAGTATTTTTAGCTATG
AATCTGAATAGCTAAAAATGCTTACATTTTAGGACGTGTGTCCAGATTTATAGCTAAAAATGTTTATATTTTGGGA
CGGAGGGAGTATAAATTAAGGGTCGATGATATCTCTCATTATATCATAGATGATGCACGGAATATTTGATTAGTCA
ACATGCATGCCTTACTCATAATATAAACCATCTTAAAAATGGATAATATATGCATGATATATATCTCTCATTATAT
CAGAACGTATATATAACATCGATTGTTTTGCACACGCTCAAAATTGTCATATTTTTTTTCTAAAAAGGAAATATAC
AACATAGTTGATTATCCTATACGTCCCTCTACAAATTAACACACATTGCTAGGGGTGAAAACGGGGCGGGTATTTC
CCACTCGCCCGCCCACCTGGCCACATTATTTTGGGACAAATTTGGGTACTCTGGACTTTTCACGGATATCAGGTCC
AAATACGGGTCTTTTTTGCGGATATGAGATCGGGTTCGGGAACGTAGTATCTGACTGATACGGATTATATGGATAT
TACCCGTATTATGTTTTTGTGAGGTAATTTTGATACTATGTATTGGTTATACATCGTATTGTACCCTATTTATTTC
CTAAGGTCCTAATAGCCTACTATAATCGTATTATGGATATATAAAATTGTTAAATAATTATTGTCTATCTGATATA
TAAGATTGTTGCTTCGACTTGATATCCAAATAAATATTCGTAACCGATAGTGTACGTACCATTTTGGTACCTATTT
GTTCCATATTTTTGCTCGACATTATCTGAGTTTGTATCCATATTCAACACTATCCGTGTTTGATCCGATTCCAATT
ATAAAATATGGGTTAGGATATGGGAAGGGTAAGATCCGACCGAACCCGACCCGTTTTCACCCCTACACGTCGCTAA
TCTACACTTCGAGGCAAAAAAAAAAGAGAGTAACAAATCAAACTACAAAAGCGCGCAATCGCATACACAATAATTA
AATTTGTATCTATCGTCACATATACAAATAAAATTTGATCTTGCATATTTGCACCACAATAAGGTAATACCTATGT
(-465bp— -451bp )
TTTCTACTTTTTAGAAAATTTATTAATAACTTTTTTTAGATAATTTATGAATAACTATTTGGACCACAGGAAATAG
ACAACGTAGTACGACACATTCTTTCCTAGGTCTTATGTACGCATGATTGTGTGTATATTCTCGCCTACGCCGACGA
CGGTGACCGGTGCATATGTTCCCAGCCGTCCTCCGCCGCGCATTGCGGTTGTGGAGGAAGGGAGCTCCACGTCTCG
CCATGGCCGTCCACAACCTGAGTTAGATCTCTAGCTACCTGATCCCCAAACCCTCTCAAAAAGATGTATATTCTTC
CTAGCACTCTGGCCCCTGGATTAGCTCAAAAATTCCTCATATATATGCTGGCTAGCTAGCTGATAGTATATACTAC
TCATAACCCATTCTTCTTCCTTACCTAGCTAG

**Fig. S6. Identification of SIP1 binding sites in the *Ehd1* promoter.**

The SIP1 binding sites are shown in red and the position is marked in the figure. The transcription start site is indicated in red and the region for EMSA is underlined.

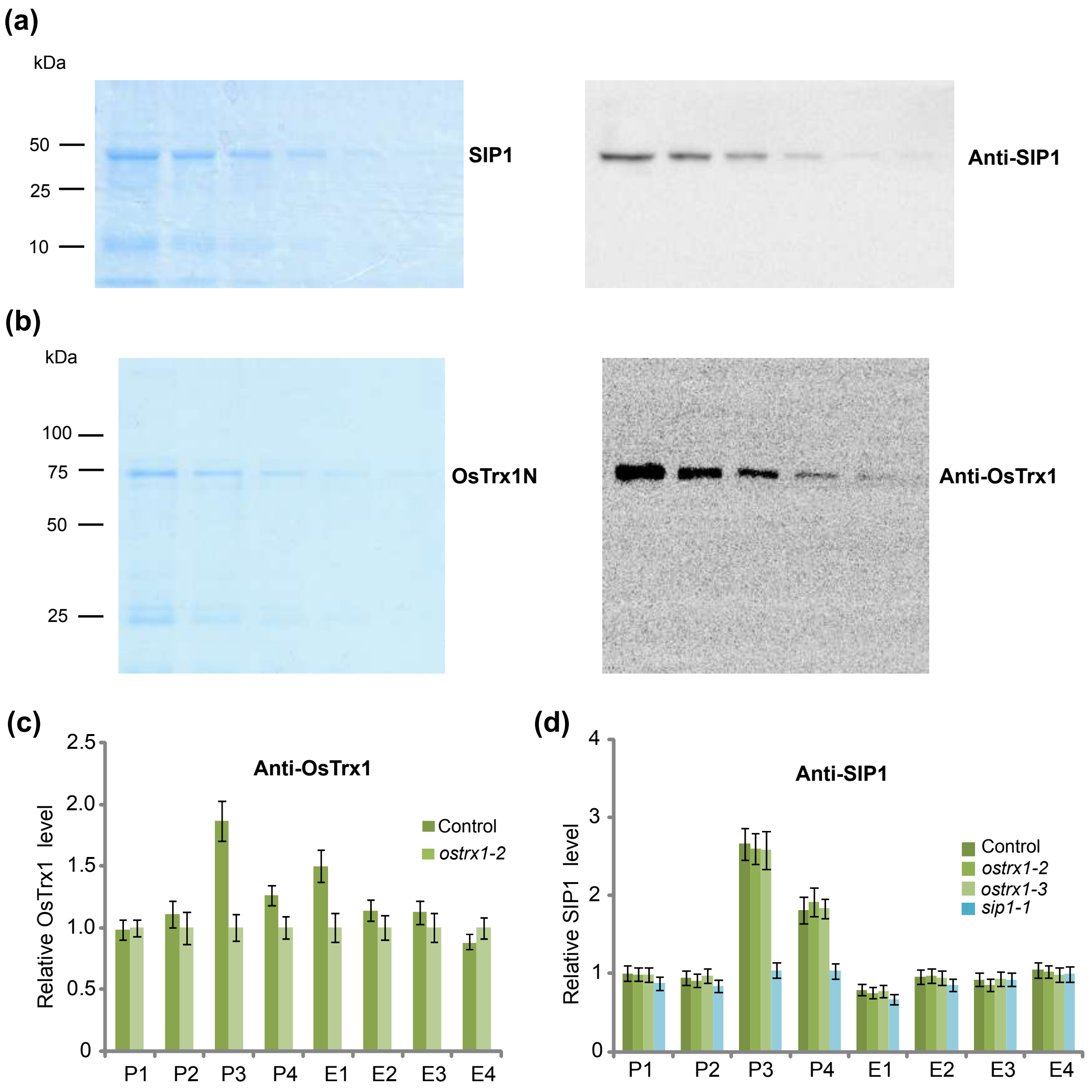


**Fig. S7. The specificity of the antibodies for SIP1 and OsTrx1.**

(a) The SIP1 protein was stained with Coomassie blue (left panel), and the protein position is indicated on the right. A specific antibody for SIP1 was generated (right panel); the specificity of the antibody was tested at a 1:1000 dilution. Molecular mass markers in kiloDaltons (kDa) are indicated on the left.

(b) The N-terminus of OsTrx1 was stained with Coomassie blue (left panel); the protein position is indicated on the right. A specific antibody for OsTrx1 was generated (right panel); the specificity of the antibody was tested at a 1:1000 dilution. Molecular mass markers in kiloDaltons (kDa) are indicated on the left.

(c) Relative OsTrx1 level in wild-type and *ostrx1-2* plants using OsTrx1 antibody. The OsTrx1 profile with OsTrx1 antibody is similar to that in transgenic lines harboring $Pro_{OsTrx1}$: *FLAG-OsTrx1* in Figure 1C. Experiments were repeated at least three times, and the data from the representative experiment shown are presented as means ± SE, *n* = 3 replicates.

(d) Relative SIP1 level in wild-type and *ostrx1-2*, *ostrx1-2*, and *sip1-1* plants using OsTrx1 antibody. Experiments were repeated at least three times, and the data from the representative experiment shown are presented as means ± SE, *n* = 3 replicates.

## Table S 1

**The average daylight in 10-day intervals in 2017 at Hefei and Lingshui, China**

**Daylight in Hefei (31°95′)**

| From | To | Average daylight |
|---|---|---|
| 21-Apr | 30-Apr | 13.26h |
| 1-May | 10-May | 13.55h |
| 11-May | 20-May | 13.79h |
| 21-May | 30-May | 13.99h |
| 1-Jun | 10-Jun | 14.15h |
| 11-Jun | 20-Jun | 14.22h |
| 21-Jun | 30-Jun | 14.23h |
| 1-Jul | 10-Jul | 14.16h |
| 11-Jul | 20-Jul | 14.03h |
| 21-Jul | 30-Jul | 13.84h |
| 1-Aug | 10-Aug | 13.59h |
| 11-Aug | 20-Aug | 13.31h |
| 21-Aug | 30-Aug | 13.02h |
| 1-Sep | 10-Sep | 12.70h |
| 11-Sep | 20-Sep | 12.37h |
| 21-Sep | 30-Sep | 12.04h |

**Daylight in Lingshui (18°50′)**

| From | To | Average day length |
|---|---|---|
| 11-Nov | 20-Nov | 11.19 |
| 21-Nov | 30-Nov | 11.07 |
| 1-Dec | 10-Dec | 10.98 |
| 11-Dec | 20-Dec | 10.93 |
| 21-Dec | 30-Dec | 10.93 |
| 1-Jan | 10-Jan | 10.97 |
| 11-Jan | 20-Jan | 11.06 |
| 21-Jan | 30-Jan | 11.18 |
| 1-Feb | 10-Feb | 11.34 |
| 11-Feb | 20-Feb | 11.51 |
| 21-Feb | 28-Feb | 11.68 |

**Table S 2. constructs and primers**

**GST-OsTrx1N**
The full length OsTrx1N cDNA was amplified using forward (5'-CCGGAATTCATGGTGATCGCGGTGGAGGGGGGC-3') and reverse (5'-CCGCTCGAGTCAAGAACTGCCCTTTTCCATGGAT-3') primers, and then cloned into pGEX-6p-1 vector with EcoRI/XhoI cohesive ends.
This vector was for pull-down assay.

**GST- OsTrx1C**
The full length OsTrx1C cDNA was amplified using forward (5'-CGCGGATCCGAAGCCAAGGAGTTTCTCTGCACA-3') and reverse (5'-CCGCTCGAGTCATTGTTGAAATAATTCACTTCTA-3') primers, and cloned into pGEX-6p-1 vector with BamHI/XhoI cohesive ends.
This vector was for pull-down assay.

**His-SIP1**
The full length SIP1 cDNA was amplified using forward (5'-CGGAATTCATGGGGAAGAAGAAGAAGCGCGTGG-3') and reverse (5'-CGGAATTCCAGGGCCATGCGTCCAGCAAGCCGA-3') primers, and cloned into pET30a vector with EcoRI cohesive ends.
This vector was for pull-down and EMSA assay.

**pGBKT7-SIP1**
The full length SIP1 cDNA was amplified using forward (5'-CGGAATTCATGGGGAAGAAGAAGAAGCGCGTGG-3') and reverse (5'-CGGAATTCCAGGGCCATGCGTCCAGCAAGCCGA-3') primers, and cloned into pGBKT7 vector with EcoRI cohesive ends.
This vector was used for yeast two-hybrid assay with S. *cerevisiae* AH109。

**pGADT7-SIP1**
The full length SIP1 cDNA was amplified using forward (5'-CGGAATTCATGGGGAAGAAGAAGAAGCGCGTGG-3') and reverse (5'-CGGAATTCCAGGGCCATGCGTCCAGCAAGCCGA-3') primers, and cloned into pGADT7 vector with EcoRI cohesive ends.
This vector was used for yeast one-hybrid assay with Y1HGold system.

**pGADT7- OsTrx1**
The full length OsTrx1 cDNA was amplified using forward (5'-GGAATTCCATATGGTGATCGCGGTGGAGGG-3') and reverse (5'-CCGCTCGAGTCATTGTTGAAATAATTCACTTCTA-3') primers, and cloned into pGADT7 vector with NdeI/XhoI cohesive ends.
This vector was used for yeast two-hybrid assay with S. *cerevisiae* AH109。

**pGADT7- OsTrx1N**

The full length OsTrx1N cDNA was amplified using forward (5'-GGAATTCCATATGGTGATCGCGGTGGAGGG-3') and reverse (5'-CCGCTCGAGTCAAGAACTGCCCTTTTCCATGGAT-3') primers, and cloned into pGADT7 vector with NdeI/XhoI cohesive ends.

This vector was used for yeast two-hybrid assay with S. *cerevisiae* AH109。

**pGADT7- OsTrx1C**

The full length OsTrx1C cDNA was amplified using forward (5'-CCGGAATTCGAAGCCAAGGAGTTTCTCTGCACA -3') and reverse (5'-CCGCTCGAGTCATTGTTGAAATAATTCACTTCTA-3') primers, and cloned into pGADT7 vector with EcoRI/XhoI cohesive ends.

This vector was used for yeast two-hybrid assay with S. *cerevisiae* AH109。

**YFP$^{C}$-OsTrx1**

The full length OsTrx1 cDNA was amplified using forward (5'-CCGCTCGAGATGGTGATCGCGGTGGAGGGGGGCTTCG-3') and reverse (5'-CCGCTCGAGTTGTTGAAATAATTCACTTCTATTGACCC-3') primers, and cloned into pUC-SPYCE vector with XhoI cohesive ends.

This vector was used for BIFC assay by injecting tobacco leaf with EHA105 or transient expression in rice protoplast。

**YFP$^{N}$-SIP1**

The full length SIP1 cDNA was amplified from using forward (5'-CGGACTAGTATGGGGAAGAAGAAGAAGCGCGTGG-3') and reverse (5'-CGGGATCCCAGGGCCATGCGTCCAGCAAGCCGA-3') primers, and cloned into pUC-SPYNE vector with SpeI/BamHI cohesive ends.

This vector was used for BIFC assay by transient expression in rice protoplast。

**PUC19-GFP-OsTrx1**

The full length OsTrx1 cDNA was amplified using forward (5'-CGGACTAGTATGGTGATCGCGGTGGAGGGGGGCTTC-3') and reverse (5'-CGGACTAGTTTGTTGAAATAATTCACTTCTATTGACCC-3') primers, and cloned into pUC19-GFP vector with SpeI cohesive ends.

This vector was used for Co-ip assay and GUS activity assay by transient expression in rice protoplast。

**PUC19-FLAG-SIP1**

The full length SIP1 cDNA was amplified using forward (5'-ACGCGTCGACATGGGGAAGAAGAAGAAGCGCGTGG-3') and reverse (5'-ACGCGTCGACCAGGGCCATGCGTCCAGCAAGCCGA-3') primers, and cloned into pUC19-FLAG vector with SalI cohesive ends.

This vector was used for Co-ip assay and GUS activity assay by transient expression

in rice protoplast。

**PUC19-HA-OsTrx1**

The full length OsTrx1 cDNA was amplified using forward (5’-ACGCGTCGACATGGGGAAGAAGAAGAAGCGCGTGG-3’) and reverse (5’-ACGCGTCGACCAGGGCCATGCGTCCAGCAAGCCGA-3’) primers, and cloned into pUC19-HA vector with SalI cohesive ends.

This vector was used for Co-ip assay and GUS activity assay by transient expression in rice protoplast。

**Pro$_{Ehd1}$:GUS**

To generate Pro$_{Ehd1}$:GUS, the Ehd1 promoter was amplified from Rice genome using forward (5’-TTCCTCAATGCATTGCCAGCCAGCC-3’) and reverse primer (5’-GATCCATTATTATAACCTAATTAGGAG-3’), and cloned into pUC19-GUS with blunt ends.

This vector was used for GUS activity assay by transient expression in rice protoplast.

**Pro$_{RFT1}$:GUS**

To generate Pro$_{RFT1}$:GUS, the RFT1 promoter was amplified from Rice genome using forward (5’- TAGTGATTCGGTGGGCGTAACTGGTA-3’) and reverse primer (5’-ATGTCAAATTAATAACCTCTAACTAA-3’), and cloned into pUC19-GUS with blunt ends.

This vector was used for GUS activity assay by transient expression in rice protoplast.

**Pro$_{Hd3a}$:GUS**

To generate Pro$_{Hd3a}$:GUS, the Hd3a promoter was amplified from Rice genome using forward (5’-AAATCGCAACACCGTAGTATAGTAC-3’) and reverse (5’-CGATCTTGCAAAAAACCCTGAAGGTT-3’) primers, and cloned into pUC19-GUS with blunt ends.

**Pro$_{EHD1}$:AbAi(-2000~ -1)**

To generate Pro$_{Ehd1}$:AbAi, the Ehd1 promoter was amplified from Rice genome using forward(5’-CCCAAGCTTATGCATTGCCAGCCAGCCTATCCAG-3’) and reverse (5’-CCGCTCGAGCTAGCTAGGTAAGGAAGAAGAATGG -3’) primers and cloned into pAbAi vector with HindⅢ and XhoI cohesive ends.

This vector was used for Y1H assay with Y1H Gold system.

**Pro$_{EHD1P1}$:AbAi(-700~ -1)**

To generate Pro$_{Ehd1P1}$:AbAi, the Ehd1 promoter was amplified from Rice genome using forward(5’-CCCAAGCTTCGACATTATCTGAGTTTGTATCCA-3’) and reverse (5’- CCGCTCGAGCTAGCTAGGTAAGGAAGAAGAATGG -3’) primers and cloned into pAbAi vector with HindⅢ and XhoI cohesive ends.

This vector was used for Y1H assay with Y1HGold system.

**Pro$_{EHD1P2}$:AbAi(-600~ 0)**

To generate Pro$_{Ehd1P2}$:AbAi, the Ehd1 promoter was amplified from Rice genome using forward(5'-CCCAAGCTTCGAACCCGACCCGTTTTCACCCCT-3') and reverse (5'- CCGCTCGAGCTAGCTAGGTAAGGAAGAAGAATGG -3') primers and cloned into pAbAi vector with HindⅢ and XhoI cohesive ends.

This vector was used for Y1H assay with Y1HGold system.

**Pro$_{EHD1P3}$:AbAi(-500~0)**

To generate Pro$_{Ehd1P3}$:AbAi, the Ehd1 promoter was amplified from Rice genome using forward(5'-CCCAAGCTTACACAATAATTAAATTTGTATCTA-3') and reverse (5'-CCGCTCGAGCTAGCTAGGTAAGGAAGAAGAATGG -3') primers and cloned into pAbAi vector with HindⅢ and XhoI cohesive ends.

This vector was used for Y1H assay with Y1HGold system.

**Pro$_{EHD1P4}$:AbAi(-400~0)**

To generate Pro$_{Ehd1P4}$:AbAi, the Ehd1 promoter was amplified from Rice genome using forward(5'-CCCAAGCTTAGAAAATTTATTAATAACTTTTTT-3') and reverse (5'-CCGCTCGAGCTAGCTAGGTAAGGAAGAAGAATGG -3') primers and cloned into pAbAi vector with HindⅢ and XhoI cohesive ends.

This vector was used for Y1H assay with Y1HGold system

.

**Pro$_{EHD1P5}$:AbAi(-2000~-500)**

To generate Pro$_{Ehd1P5}$:AbAi, the Ehd1 promoter was amplified from Rice genome using forward(5'-CCCAAGCTTATGCATTGCCAGCCAGCCTATCCAG-3') and reverse (5'- CCGCTCGAGTATGCGATTGCGCGCTTTTGTAGTT-3') primers and cloned into pAbAi vector with HindⅢ and XhoI cohesive ends.

This vector was used for Y1H assay with Y1HGold system.

**Pro$_{EHD1P6}$:AbAi(-700~-451)**

To generate Pro$_{Ehd1P6}$:AbAi, the Ehd1 promoter was amplified from Rice genome using forward(5'-CCCAAGCTTCGACATTATCTGAGTTTGTATCCAT-3') and reverse (5'- CCGCTCGAGTCAAATTTTATTTGTATATGTGACG-3') primers and cloned into pAbAi vector with HindⅢ and XhoI cohesive ends.

This vector was used for Y1H assay with Y1HGold system.

**Pro$_{EHD1P7}$:AbAi(-451~-1)**

To generate Pro$_{Ehd1P7}$:AbAi, the Ehd1 promoter was amplified from Rice genome using forward(5'-CCCAAGCTTATCTTGCATATTTGCACCACAATAA-3') and reverse (5'- CCGCTCGAGCTAGCTAGGTAAGGAAGAAGAATGG -3') primers and cloned into pAbAi vector with HindⅢ and XhoI cohesive ends.

This vector was used for Y1H assay with Y1HGold system.

. **Pro$_{EHD1P8}$:AbAi(-700~-475)**

To generate Pro$_{Ehd1P8}$:AbAi, the Ehd1 promoter was amplified from Rice genome

using forward(5’-CCCAAGCTTCGACATTATCTGAGTTTGTATCCAT-3’) and reverse (5’- CCGCTCGAGGATAGATACAAATTTAATTATTGTG -3’) primers and cloned into pAbAi vector with HindIII and XhoI cohesive ends.
This vector was used for Y1H assay with Y1HGold system.

**Pro$_{EHD1P9}$:AbAi(-475~-1)**
To generate Pro$_{Ehd1P9}$:AbAi, the Ehd1 promoter was amplified from Rice genome using forward(5’-CCCAAGCTTCGTCACATATACAAATAAAATTTGA-3’) and reverse primer (5’- CCGCTCGAGCTAGCTAGGTAAGGAAGAAGAATGG -3’) primers and cloned into pAbAi vector with HindIII and XhoI cohesive ends.
This vector was used for Y1H assay with Y1HGold system.

.
**Pro$_{EHD1P10}$:AbAi(-465~-1)**
To generate Pro$_{Ehd1P10}$:AbAi, the Ehd1 promoter was amplified from Rice genome using forward(5’- CCCAAGCTTACAAATAAAATTTGATCTTGCATAT-3’) and reverse (5’- CCGCTCGAGCTAGCTAGGTAAGGAAGAAGAATGG -3’) and cloned into pAbAi vector with HindIII and XhoI I cohesive ends.
This vector was used for Y1H assay with Y1HGold system.

**Pro$_{EHD1P11}$:AbAi(-700~-460)**
To generate Pro$_{Ehd1P11}$:AbAi, the Ehd1 promoter was amplified from Rice genome using forward(5’-CCCAAGCTTCGACATTATCTGAGTTTGTATCCAT-3’) and reverse (5’- CCGCTCGAGATTTGTATATGTGACGATAGATACA -3’) primers and cloned into pAbAi vector with HindIII and XhoI cohesive ends.
This vector was used for Y1H assay with Y1HGold system.

**pHUN4c12-OsTRX1-2**
To generate pHUN4c12-OsTRX1-2 vector for CRISPR/cas9, 20bp target(5’-GCCGCCGCCGTAAGAAGCCG-3’) of sense strand was chosen. pHUN4c12 vector was digested with BsaI. Poly-nucleotide 5’-ggcaGCCGCCGCCGTAAGAAGCCG-3’ and 5’-aaacCGGCTTCTTACGGCGGCGGC-3’ were used annealing to form Complementary double stand DNA with the same cohesive ends and then be cloned into the vector.
This vector was used to generate *ostrx1-2* cas9 mutant.

**pHUN4c12-OsTRX1-3**
To generate pHUN4c12-OsTRX1-3 vector for CRISPR/cas9, 20bp target(5’-TGGTACAAGGGTTCTATCAC-3’) of sense strand was chosen. pHUN4c12 vector was digested with BsaI. Poly-nucleotide 5’-ggcaTGGTACAAGGGTTCTATCAC-3’ and 5’-aaacGTGATAGAACCCTTGTACCA-3’ were were used annealing to form complementary double stand DNA with the same cohesive ends and then be cloned into the vector.

This vector was used to generate *ostrx1-3* cas9 mutant.

**pHUN4c12-Ehd1-11**

To generate pHUN4c12-OsTRX1-3 vector for CRISPR/cas9, 20bp target(5'-CGATCCTCACAGGTTTTAGC-3') of antisense strand was chosen. pHUN4c12 vector was digested with BsaI. Poly-nucleotide 5'-ggcaCGATCCTCACAGGTTTTAGC-3' and 5'-aaacGCTAAAACCTGTGAGGATCG-3' were were used annealing to form complementary double stand DNA with the same cohesive ends and then be cloned into the vector.

This vector was used to generate *ehd1-11* cas9 mutant

**pHUN4c12-SIP1-1**

To generate pHUN4c12-OsTRX1-3 vector for CRISPR/cas9, 20bp target(5'-GATCCTCGTGCAGCACCAGA-3') of sense strand was chosen. pHUN4c12 vector was digested with BsaI. Poly-nucleotide 5'-ggcaGATCCTCGTGCAGCACCAGA -3' and 5'-aaacTCTGGTGCTGCACGAGGATC -3' were were used annealing to form complementary double stand DNA with the same cohesive ends and then be cloned into the vector.

This vector was used to generate *sip1-1* cas9 mutant

**pHUN4c12-SIP1-2**

To generate pHUN4c12-OsTRX1-3 vector for CRISPR/cas9, 20bp target(5'-TGACGGACTCCTTGTGGACC-3') of antisense strand was chosen. pHUN4c12 vector was digested with BsaI. Poly-nucleotide 5'-ggca TGACGGACTCCTTGTGGACC-3' and 5'-aaacGGTCCACAAGGAGTCCGTCA-3' were were used annealing to form complementary double stand DNA with the same cohesive ends and then be cloned into the vector.

This vector was used to generate *sip1-2* cas9 mutant

**pHUN4c12-SIP1-3**

To generate pHUN4c12-OsTRX1-3 vector for CRISPR/cas9, 20bp target(5'-GCTTTGCATTGGGAACCCTG-3') of antisense strand was chosen. pHUN4c12 vector was digested with BsaI. Poly-nucleotide 5'-ggca GCTTTGCATTGGGAACCCTG -3' and 5'-aaac CAGGGTTCCCAATGCAAAGC-3' were were used annealing to form Complementary double stand DNA with the same cohesive ends and then be cloned into the vector.

This vector was used to generate *sip1-3* cas9 mutant

**$Pro_{35S}$: OsTrx1**

To generate $Pro_{35S}$:OsTrx1, the OsTrx1 cDNA was amplified using forward (5'-GGGGTACCATGGTGATCGCGGTGGAGGGGGGCTTC-3') and reverse primer (5'-GGGGTACCTTGTTGAAATAATTCACTTCTATTGACCC-3'), and then cloned into pCAMBIA1300 with KpnI cohesive ends.

This vector was transferred into *atx1-1* mutant to get complementary lines.

**Pro$_{OsTrx1}$: OsTrx1-FLAG**

To generate Pro$_{OsTrx1}$:FLAG- OsTrx1, the 3kb length OsTrx1 promoter was amplified from Rice genome using forward (5'-CGGGATCCAAGGTTCCACTCCAATGACCAAGGC-3') and reverse primer (5'-CGGAATTCGGCTGGGCTGGCTAGGGTTTCGGCT-3'), and cloned into pCAMBIA1300FLAG with blunt ends. The OsTrx1 cDNA was amplified from an Rice first strand cDNA pool using forward primer (5'-GGGGTACCATGGTGATCGCGGTGGAGGGGGGCTTC-3') and reverse primer (5'-GGGGTACCTTGTTGAAATAATTCACTTCTATTGACCC-3'), cloned into Pro$_{OsTrx1}$:pCAMBIA1300FLAG with with KpnI cohesive ends. Flag tag was down stream of OsTrx1 C terminal.

This vector was transferred into *ostrx1-2* mutants to get complementary lines.

**Genotyping primers for Cas9 mutants**

LP1 (5'- TGGTTGGTGTCCGTTAGACTCGTCGA-3')

RP1(5'- GTCGTGCCCCTCTCTAGAGATAATGA-3')

LP1 RP1 are located in ORF of Cas9 coding sequence and were used for genotyping cas9 mutants in T1 generation to find seedlings without CRISP/Cas9 constucts.

*ostrx1-2*

LP2 (5'-CCGGAATTCATGGTGATCGCGGTGGAGGGGGGC-3')

RP2 (5'-CGGGATCCTTCCGAGGCAAGCGTAGACAGATCC-3')

LP2 was used as sequencing primer of the PCR product, homozygous shows single peak but mutated sequence in the target region from the sequencing results.

*ostrx1-2*

LP3 (5'-CGGGATCCACTGGCCTTTGTGGGATTAGG-3')

RP3 (5'-GGTTAAGGTCCTCTGATTCGCCATCATC-3')

LP3 was used as sequencing primer of the PCR product, homozygous shows single peak but mutated sequence in the target region from the sequencing results.

*sip1-1 and sip1-2*

LP4 (5'-CGGAATTCATGGGGAAGAAGAAGAAGCGCGTGG-3')

RP4 (5'-CTGTCACCACCAAGATGCCAACCCAA-3')

LP4 was used as sequencing primer of the PCR product, homozygous shows single peak but mutated sequence in the target region from the sequencing results.

*sip1-3*

LP5 (5'- CAACTGCCGTGTTTGCGTATTTGCGG-3')

RP5 (5'-CTGTCACCACCAAGATGCCAACCCAA-3')

LP5 was used as sequencing primer of the PCR product, homozygous shows single peak but mutated sequence in the target region from the sequencing results.

*ehd1-11*

LP6 (5’- ATATGCCGTCACAATGGGCTGCTCTAG-3’)
RP6 (5’- GTAGTTGACCATGTCATCTCTCACCTC-3’)
LP6 was used as sequencing primer of the PCR product, homozygous shows single peak but mutated sequence in the target region from the sequencing results.

**qPCR primers**
*UBQ1* (Os03g0234200)
forward primer (5’- TGAAGACCCTGACTGGGAAG-3’)
and reverse primer (5’- CACGGTTCAACAACATCCAG -3’)

*Ehd1*(Os10g0463400)
forward primer (5’-GTTGCCAGTCATCTGCAGAA-3’)
and reverse primer (5’-GGATGTGGATCATGAGACAT-3’)

*RFT1* (Os06g0157500)
forward primer (5’-TGGGTTAGCTGACCTAGATTCAAA-3’)
and reverse primer (5’-GCCAACCACAAGAGGATCGT-3’)

*Hd3a* (Os06g0157700)
forward primer (5’-AGCCCAAGTGACCCTAACCT-3’)
and reverse primer (5’-GTTGTAGAGCTCGGCGAAGT-3’)

*OsMADS50* (Os03g0122600)
forward primer (5’-AAAGCTGACGCTGATGGTTTG-3’)
and reverse primer (5’-GTTTCGACATCCATGTTGTC-3’)

*OsMADS51*(Os01g0922800)
forward primer (5’-GACGAGTCGGATGCTAATCA-3’)
and reverse primer (5’-CTGCTCCTACTCCCTTCACC-3’)

*OsMADS56*(Os10g0536100)
forward primer (5’- CTGGTGGCTAACCACATGAC-3’)
and reverse primer (5’- CAGATCAGTCTCCACGTCCA-3’)

*Hd1*(Os06g0275000)
forward primer (5’- AACCAAGATCGGCAGTATGG-3’)
and reverse primer (5’- GATTGATTGCTCCAGCAGGT-3’)

*Ghd7*(Os07g0261200)
forward primer (5’- CATATTGTGGGAGCACGTT-3’)
and reverse primer (5’- ATCTGAACCATTGTCCAAGC-3’)

*Ehd3*(Os08g0105000)

forward primer (5’- AAGACAAGGATGATGACCAG-3’)
and reverse primer (5’- TACCATCACCTTCAGAATCC-3’)

*Ehd2*(Os10g0419200)
forward primer (5’- AGCTCACCACCACTGTTCC-3’)
and reverse primer (5’- GTGAGCTGCTTGGTGTTGTT-3’)

*Ehd4*(Os03g0112700)
forward primer (5’- GATGGAGCAAAGTTGTGGAA-3’)
and reverse primer (5’- GCATGTGGATAAAGCAATGG-3’)

*EL1(*Os03g0793500)
forward primer (5’- TGGGTAAAGGTGGATTTGGT-3’)
and reverse primer (5’- CTTTACTGCTTCGGTGCTCA-3’)

*OsLFL1*(Os01g0713600)
forward primer (5’- GATGGTATGGACATGGCAAA-3’)
and reverse primer (5’- ACTTTGGGTTGGGAATCAAG-3’)

**ChIP PCR primers**

***Ehd1***
Region P1
forward primer (5’- TTGGCTGAGCTGAGGGAAAT-3’)
and reverse primer (5’- AGGCACCATGAAAGAGAACA-3’)

Region P2
forward primer (5’- GCGGGTATTTCCCACTCG-3’)
and reverse primer (5’- ACCCGATCTCATATCCGCAA-3’)

Region P3
forward primer (5’- GACCGAACCCGACCCGTTT-3’)
and reverse primer (5’- GTGTATGCGATTGCGCGCTT-3’)

Region P4
forward primer (5’- AGCTAGCTGGAGGAGGAACA-3’)
and reverse primer (5’- ATCACCAAAGCTAGCCTGCC-3’)

Region E1
forward primer (5’- CACCGAGAGCTGTGGCCTTA-3’)
and reverse primer (5’- AGAAGTAAATCTTCCATGACTGACA-3’)

Region E2
forward primer (5’- GTCTTGGAGGGTAGTGCAGA-3’)

and reverse primer (5’- TGGAGTATGGATCGCCCATT-3’)

Region E3
forward primer (5’- AACCCCATCTACAACTGGCT-3’)
and reverse primer (5’- AACACTTCACTAGAGCAGCCC-3’)

Region E4
forward primer (5’- ATTTGCAGAGGCAGTTCCAAAG-3’)
and reverse primer (5’- CATCTTGCAATGAGCCACTGA-3’)

***RFT1***
Region P1
forward primer (5’- AATCAACCCAAGCCGCTCTA-3’)
and reverse primer (5’- ACCAGGACAGAGCAAGACTG-3’)

Region P2
forward primer (5’- GGCCGGGAATAATGTCTAGC-3’)
and reverse primer (5’- TGCATGCATATGTGGAGGAG-3’)

Region P3
forward primer (5’- TGTCTCGAAATCGCCTCTGT-3’)
and reverse primer (5’- CACTTTGTTGTGCCCGGTAG-3’)

Region P4
forward primer (5’- GTGTCTTGGGTTGGTACTGC-3’)
and reverse primer (5’- GCTAGCCAAACAGTGACAGG-3’)

Region E1
forward primer (5’- AATGACATGAGGACGTTCTACAC-3’)
and reverse primer (5’- ATTAAATAACTTCTGGTGGGTCT-3’)

Region E2
forward primer (5’- GCCCAAGCAACCCTAACC-3’)
and reverse primer (5’- CCAGCCTACAGACAGACAAAGA-3’)

Region E3
forward primer (5’- TTTCGTCAGATTTGAAGGATAGGG-3’)
and reverse primer (5’- TTTTGAGGGGATCACCACCC-3’)

***Hd3a***
Region P1
forward primer (5’- TTTCCAACGTTAGCATCCAC-3’)
and reverse primer (5’- TTTATACCATTTCCGTACCGTTAG-3’)

Region P2
forward primer (5’- TCCCATCTCTCCTCACTTCA-3’)
and reverse primer (5’- AGTCGACGTCGTAGTTGGAA-3’)

Region P3
forward primer (5’- GCAGTACACTGACCGAGCTA-3’)
and reverse primer (5’- CTGGATCGAGCTGTGGTTG-3’)

Region E1
forward primer (5’- TTGTGGTTGGTAGGGTTG-3’)
and reverse primer (5’- AAGGGTGTAGAATGTCCTCATG-3’)

Region E2
forward primer (5’- GATATGTCGGCTTGGGCTTGG-3’)
and reverse primer (5’- CGAATCATCCCGTCACTATCTT-3’)

Region E3
forward primer (5’- AATAAGAAGAATGATCGTCAA-3’)
and reverse primer (5’- ACACATGAAGAAGAAATGTTC-3’)

**Ghd7**
Region P1
Forward primer (5’- TATACCACCACCCACACACC-3’)
Reverse primer (5’- GTTGGTCACGCTTAGTGACG-3’)

Region P2
Forward primer (5’- TCGGTCCAGTCAAGACCAAG-3’)
Reverse primer (5’- ACACATCGGCAGAGGTTGAG-3’)

Region P3
Forward primer (5’- AAGGTCTACCACAAGCATAAAG -3’)
Reverse primer (5’- AGCTAATCCCTTGCTGAAATTTAAC -3’)

Region P4
Forward primer (5’- GGATCGAGGTCCTGGGTTCG-3’)
Reverse primer (5’- ACCGTCAGATTCATCTCAAGATTC-3’)

Region E1
Forward primer (5’- CCAGCAGCCGGAGAAGGATG-3’)
Reverse primer (5’- CTCGGCGGGAAGACGAAGG-3’)

Region E2
Forward primer (5’- GGCCGTGTGTGGGATGTGAT-3’)

Reverse primer (5’- AGACAGAGGCTGGATCCGTA-3’)

Region E3
Forward primer (5’- TCACGGACTCACAGAGTGTGC-3’)
Reverse primer (5’- GCATACATCGATCGGCCACCA-3’)

Region E4
Forward primer (5’- ATGGTGGAGAGGGAGGCCAA-3’)
Reverse primer (5’- GCCTCATCTCGGCATAGGCT-3’)

**EMSA probes**
***Ehd1***
WT
forward primer (5’- ACATATACAAATAAAATTTGATCTTGC-3’)
and reverse primer (5’- GCAAGATCAAATTTTATTTGTATATGT-3’)

mut1
forward primer (5’- ACATATACAAATAAAATTctcTCTTGC-3’)
and reverse primer (5’- GCAAGAgagAATTTTATTTGTATATGT-3’)

mut2
forward primer (5’- ACATATACAAATAAAcccTGATCTTGC-3’)
and reverse primer (5’- GCAAGATCAgggTTTATTTGTATATGT-3’)

mut3
forward primer (5’- ACATATACAAATcccATTTGATCTTGC-3’)
and reverse primer (5’- GCAAGATCAAATgggATTTGTATATGT-3’)

mut4
forward primer (5’- ACATATACAcccAAAATTTGATCTTGC-3’)
and reverse primer (5’- GCAAGATCAAATTTTgggTGTATATGT-3’)

mut5
forward primer (5’- ACATATctcAATAAAATTTGATCTTGC-3’)
and reverse primer (5’- GCAAGATCAAATTTTATTgagATATGT-3’)

mut6
forward primer (5’- ACATATACAAATAAAATTTGActcTGC-3’)
and reverse primer (5’- GCAgagTCAAATTTTATTTGTATATGT-3’)

mut7
forward primer (5’- ACAcccACAAATAAAATTTGATCTTGC-3’)
and reverse primer (5’- GCAAGATCAAATTTTATTTGTgggTGT-3’)
